# Topography: A Biophysical Approach to Direct the Fate of Mesenchymal Stem Cells in Tissue Engineering Applications

**DOI:** 10.3390/nano10102070

**Published:** 2020-10-20

**Authors:** Xingli Cun, Leticia Hosta-Rigau

**Affiliations:** DTU Health Tech, Centre for Nanomedicine and Theranostics, Technical University of Denmark, Nils Koppels Allé, Building 423, 2800 Kgs. Lyngby, Denmark; xincu@dtu.dk

**Keywords:** biomaterials, differentiation, mesenchymal stem cells, scaffolds, tissue engineering, topography

## Abstract

Tissue engineering is a promising strategy to treat tissue and organ loss or damage caused by injury or disease. During the past two decades, mesenchymal stem cells (MSCs) have attracted a tremendous amount of interest in tissue engineering due to their multipotency and self-renewal ability. MSCs are also the most multipotent stem cells in the human adult body. However, the application of MSCs in tissue engineering is relatively limited because it is difficult to guide their differentiation toward a specific cell lineage by using traditional biochemical factors. Besides biochemical factors, the differentiation of MSCs also influenced by biophysical cues. To this end, much effort has been devoted to directing the cell lineage decisions of MSCs through adjusting the biophysical properties of biomaterials. The surface topography of the biomaterial-based scaffold can modulate the proliferation and differentiation of MSCs. Presently, the development of micro- and nano-fabrication techniques has made it possible to control the surface topography of the scaffold precisely. In this review, we highlight and discuss how the main topographical features (i.e., roughness, patterns, and porosity) are an efficient approach to control the fate of MSCs and the application of topography in tissue engineering.

## 1. Introduction

Stem cells (SCs) can differentiate into several types of cells and, as such, they have the capacity to repair injured parts of organs and tissues, thus holding a lot of potential in regenerative medicine.

In vivo, self-renewal (the maintenance of potency), proliferation, and differentiation toward a particular cell lineage, are regulated by the SCs but also by their niche (their specific microenvironment). During tissue development, SCs fate is influenced not only by biochemical factors (in the form of growth factors, low molecular weight chemicals, hormones, etc.) but also by the biophysical cues of their extracellular matrix (ECM) microenvironment [1,2,3]. The ECM is a key component since, to undergo fundamental biological processes, cells must adhere to the underlying ECM where they receive and respond to complex molecular signals [4]. Such biophysical cues located within the ECM can be classified into surface topography, matrix stiffness and mechanical forces [5,6,7]. In particular, the effect of topography on the SCs fate was reported as early as 1964 by Curtis et al. First, the influence of topographical features in the micron range was identified and, since then, the effect of microtopography onto the cells fate has been thoroughly studied [2,3,8]. However, the ECM that cells are exposed to, also displays nanometer range features. Thanks to the recent advances in nanofabrication techniques [3,9,10], it has been also possible to demonstrate how engineered substrates with analogous nanoscale features to those of the natural ECM, are able to direct cell behavior. For example, by manipulating nanoscale topography alone, it has been possible to elicit different responses from several cell types including, osteoblasts (OBs), or mesenchymal stem cells (MSCs) [3,11,12,13,14,15].

However, despite the enormous potential that SCs hold, their use in tissue engineering (TE) is still very limited as a result of their low differentiation efficiency and the difficulties associated with guiding their differentiation towards a specific cell lineage. Growth factors and other smaller chemical compounds can effectively promote SCs differentiation; however, making use of biochemical cues has its limitations and risks. For example, supra-physiological dosages need to be administered to obtain a desired phenotype. This results in high costs, as well as potential off-target effects and challenges in attaining optimal release kinetics. Additionally, upon removal of such biochemical cues as often occurs following in vivo transplantation [16], the long-term maintenance of the SCs differentiation is rapidly lost [17]. What is more, providing the required induction factors in vivo is rather challenging. It involves the design of implants that can incorporate a combination of molecules, as well as the ability to release such chemicals with controlled manner in terms of dose and kinetics over extended periods (e.g., days or even weeks) [18].

Importantly, SCs also respond to biophysical stimuli within their surrounding ECM. Therefore, to address the limitations of the soluble factors, biomaterials-based approaches have emerged as a powerful tool to modulate the proliferation, self-renewal and differentiation of SCs into multiple lineages. Thanks to the advent of micro- and nanofabrication techniques, scaffolds mimicking the structural complexity of the ECM, including its specialized textured topography both at the micro- and nano-range, can now be fabricated [17,19]. In general, such biophysical stimuli are introduced by modifying the substrate/surface with cell-matrix interactions which will ultimately influence the mechanics of the cell cytoskeleton and, in turn, the expression of genes and proteins [20,21,22]. What is more, biophysical factors, such as topography, can be applied continuously with their effects propagating faster than the diffusion of chemical entities [18,23].

While a variety of SCs have been investigated for biomedical applications, MSCs are of particular interest [22]. MSCs are multipotent stromal SCs able to differentiate into several cell types, including adipocytes, OBs, chondrocytes; therefore, they can form fat [24], bone [25], and cartilage tissues [22,26]. Additionally, MSCs have excellent accessibility, versatility and low risk of teratoma formation [22]. Since they can be extracted from adult tissue, they are free from ethical concerns as compared to, for example, embryonic SCs. Furthermore, MSCs also display proangiogenic capabilities, immune-regulatory and anti-inflammatory potential.

**Scheme 1:**
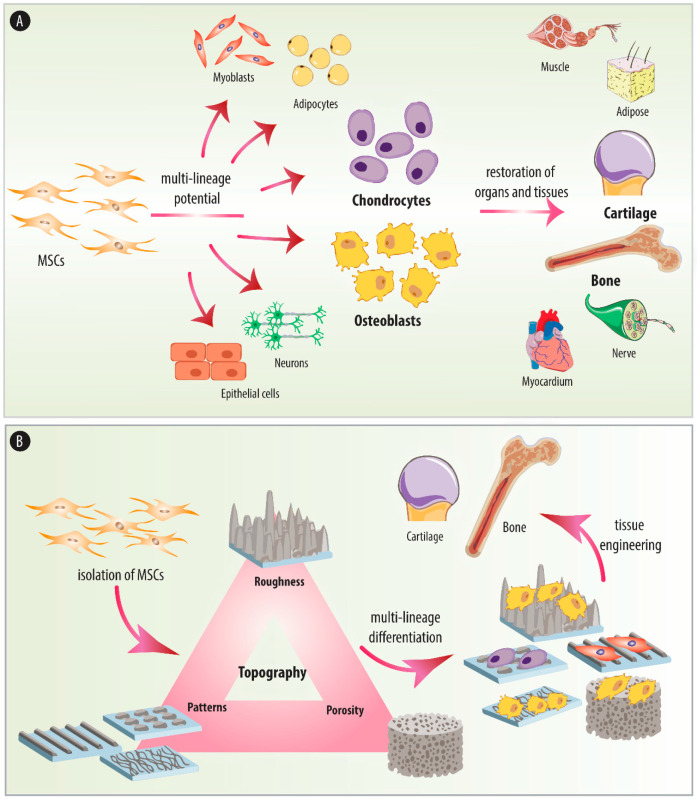
(**A**) Schematic diagram of the multilineage differentiation of mesenchymal stem cells (MSCs) and their application in tissue engineering. (**B**) The topographical properties (roughness, patterns, porosity) of the scaffolds influence the cell lineage decisions of MSCs and their application in tissue engineering. In this review, we focus on how the roughness, patterns, and porosity of biomaterials-based scaffolds direct the fate of MSCs and their application in bone and cartilage tissue engineering.

In this review, we discuss several prior studies regarding the application of topography to regulate the differentiation of MSCs. Different biomaterials-based scaffolds with control over the substrate roughness, patterns and porosity are evaluated in terms of their reported ability to either maintain the MSCs self-renewal abilities or to direct them to the cell lineage of choice. The overview of the topics covered in this review is presented in Scheme 1.

We start by providing an overview of the isolation from different tissues and characterization in terms of the differentiation potential of MSCs. The different therapeutic applications of MSCs will be also discussed. Next, we review the possible mechanisms behind the cell-surface topography interactions. The main part of this Review will be devoted to discuss how the main topographical features (i.e., roughness, surface patterns and porosity) of the biomaterials-based scaffolds control the cell-fate decisions of MSCs. Emphasis will be put in the examples examining the potential of MSCs and topography in the context of TE and the associated challenges.

## 2. Origin and Relevance of MSCs

Being the most multipotent adult SCs in the human body, MSCs have attracted a lot of interest [27]. These spindle-shaped type of cells reside in the stromal compartment of bone marrow (BM) and were identified in 1966 in a study conducted by Friedenstein and Petrakova [27,28]. In 1991, this kind of BM-isolated cells were officially named “mesenchymal stem cells (MSCs)” [29]. Interestingly, in Caplan’s latest work, he proposed to change the name of “mesenchymal stem cells” to “medicinal signaling cells” to emphasize their therapeutic effects after transplantation, which is based on the homing of MSCs to the injured or diseased site where they secrete cocktails of proteins to modulate the immune response [30]. To date, several names have been proposed for this type of cells which include: medicinal signaling cells, mesenchymal stromal cells, multipotent stromal cells, mesodermal stem cells or marrow stromal cells [30,31]. However, although the name of “mesenchymal stem cells” has raised some controversy regarding the stemness of MSCs, it is still the most commonly used name and we decide to use this name throughout this manuscript [31,32].

MSCs are an excellent cell source for tissue regeneration since they can be isolated from several adult tissues, such as BM, but also adipose tissue, where they can be easily extracted in with a minimally invasive procedure [33]. Importantly, their multipotency allows them to differentiate towards multiple cell types of the mesodermal lineage, including adipocytes, osteocytes, and chondrocytes. MSCs also circumvent complications associated with immune rejection since they can be directly extracted from a patient, induced to differentiate into a specific cell lineage to be transplanted back into the same patient [27,34].

As a result of the abovementioned properties, MSCs have attracted a lot of attention and pre-clinical and clinical studies have already demonstrated their therapeutic value [27].

### 2.1. Isolation and Characterization

MSCs can be isolated from many and rich sources. Tissues where MSCs can be extracted from include BM, adipose, synovium tissues and also blood from the umbilical cord (UC) [27,33]. The protocols to isolate the extracted MSCs involve a digestion step. Next, the cells are cultured for three to five days, the non-adherent cells discarded and the remaining adherent cells (the MSCs) are cultured to the desired passage [33]. However, since MSCs are obtained from donors, their quality differs from donor to donor and by factors, such as aging or other disorders [27,35,36]. Therefore, it is important to thoroughly characterize them to assure their quality [27]. Since MSCs do not have one specific identification marker, three criteria were stablished in 2006 by the International Society for Cellular Therapy [37]. First, the cells must be capable to adhere to plastic when cultured in standard conditions. Secondly, the cells’ surface must be positive (>95%) for particular markers, such as cluster of differentiation (CD)105, CD90, and CD73, and negative (<2%) for other markers, such as CD34, CD45, CD14, or CD79α, CD19, CD11b, and surface molecules of the human leukocyte antigen–DR isotype (HLA-DR), which is class cell surface receptor present in professional antigen-presenting cells. Thirdly, the cells must have the ability of in vitro differentiation into chondrocytes, adipocytes and OBs.

Since MSCs were first isolated from BM, BM-derived MSCs (BM-MSCs) are, so far, the most studied and thoroughly characterized MSCs. Additionally, they are regarded as the standard MSCs and used for comparison with MSCs derived from other sources. The different tissues and organs where MSCs can be extracted from show different advantages and disadvantages [33,38,39]. For example, while the extraction of MSCs from adipose tissue involves a minimally invasive procedure, UC-derived MSCs (UC-MSCs) can be harvested in a painless method [40]. Additionally, UC-MSCs renew faster than their BM-derived counterparts [41]. Lastly, an alternative approach to obtain MSCs is to derive them from induced pluripotent stem cells (iPSCs). iPSCs-derived MSCs (iPSC-MSCs) have shown similar morphology and antigen profile to BM-MSCs but have also demonstrated enhanced survival following transplantation [27,38,42].

### 2.2. Differentiation Potential

The ability to differentiate is the key feature of MSCs. However, the different tissue sources where MSCs can be obtained from, have an effect on their proliferation and differentiation potential [33]. For example, BM-MSCs have shown enhanced ability to differentiate into the osteogenic and chondrogenic lineages [43]. In contrast, MSCs derived from synovium tissues (S-MSCs) display enhanced proliferation and chondrogenic differentiation than their adipose-derived counterparts (AD-MSCs) [33,44]. UC-MSCs show several advantages over MSCs derived from other tissues, including the ability to be cultured for longer periods, large-scale expansion, and higher anti-inflammatory effects [33,45]. Therefore, depending on the application in mind, MSCs will be derived from one tissue or another.

### 2.3. Therapeutic Applications

MSCs have been broadly studied and applied in TE and regenerative medicine for the reconstruction of several tissues, including bone, cartilage, or musculoskeletal tissues.

Due to their ability to differentiate into OBs, MSCs have been widely evaluated in the context of bone regeneration. Specifically, BM-MSCs, UC-MSCs, and AD-MSCs have been extensively studied for bone TE applications. While BM-MSCs have been so far the most used type for OBs differentiation [33], UC-MSCs are also a very interesting cell type due to their angiogenic potential favoring the formation of neovasculature and promoting blood supply during the process of bone regeneration [46]. AD-MSCs are also a relevant cell type for bone TE applications since they can be isolated in good quantity and high purity with a minimally invasive procedure (lipoaspirate) [43,47]. The ability of MSCs to repair bone defects has been widely investigated both in in vitro and in vivo studies, with some of the approaches being already evaluated in clinical trials [48]. For example, biomaterials-based scaffolds pre-seeded with MSCs have been employed by dentists for the regeneration of alveolar cleft and jaw defects and for the augmentation of maxillary sinus [33,49,50,51]. Local implantation of MSCs with or without scaffold into non-human tubular bone defects has also been evaluated in clinical trials with the outcome demonstrating the safety and efficiency of such a procedure [52]. For example, patients with distal tibial fractures were injected with BM-MSCs in the fracture site, and the results showed a median time to union of 1.5 months as compared to 3 months for the control group [33,52].

Owing to their ability to differentiate into chondrocytes, MSCs have also been evaluated in the context of cartilage repair [33]. BM-MSCs, AD-MSCs but also synovium-derived MSCs (S-MSCs) [53] have been broadly studied in this context [53,54]. While BM-MSCs have shown the best results in terms of chondrogenic differentiation both in vitro and in vivo; S-MSCs have demonstrated enhanced proliferation [33,53]. Although the first clinical trial employing MSCs to treat a cartilage defect was already conducted in 1994, currently, there are no commercially available products for cartilage reconstruction using MSCs-based therapies [33,55]. Implants of expanded autologous MSCs have been used in patients with defects in knee cartilages demonstrating both the safety and the ability to alleviate some symptoms of such approaches [56,57]. However, so far, reparation of cartilage defects through MSCs-based therapy still remains to be demonstrated [33]. Due to their ability to differentiate into chondrocytes, MSCs have been explored for the regeneration of intervertebral disks. Specifically, BM-MSCs have been implanted into patients with lumbar disc degeneration demonstrating better prognosis as compared to the current treatments, such as spinal fusion or total disc replacement [58]. Such a procedure has also resulted in decreased pain, while also highlighting the safety and feasibility of intradiscal BM-MSCs therapy [59].

More recently, MSCs-based therapies have been evaluated to regenerate other musculoskeletal tissues, such as meniscus, tendons, or ligaments [33]. Clinical trials where MSCs have been administered for meniscal regeneration in osteoarthritis patients have been already conducted, and positive outcomes in terms of the safety of the procedure and pain relief have been obtained [60]. However, despite these encouraging results, there is still a lack of evidence regarding the ability of MSCs to repair and form tissues similar to the meniscus [61]. MSCs have also been explored in the context of tendon injury since they can differentiate into the tendogenic lineage following mechanical stimulation. Tendogenic differentiation under the influence of uniaxial cyclic stretching has been reported both in vitro and in vivo, with some reports showing the ability of MSCs in delaying lesion progression [62].

MSCs have also neuronal differentiation potential, making them a good cell source to repair damaged neurons of the central nervous system [63]. Clinical studies have already been conducted in patients with spinal cord and/or brain injury [64,65]. The outcome of these studies has demonstrated the feasibility and safety of such a procedure, however, with limited efficacy. MSCs have also been employed for the treatment of neurological disorders, including multiple sclerosis, ischemic stroke, and Parkinson’s disease [66,67,68]. The different clinical trials have shown diverse degrees of remission which have been attributed to the immunomodulatory and neuroprotective properties of MSCs [33].

Administration of MSCs has been used to improve cardiac function in the context of several cardiovascular diseases (e.g., myocardial infarction or cardiomyopathy) [69]. Clinical trials have demonstrated the ability of MSCs to improve myocardial perfusion and function at the ischaemic region, which is attributed to their ability to differentiate into cardiomyocytes [33,69].

Liver regeneration with MSCs has also been considered due to their ability to exert potent immunosuppressive and anti-inflammatory effects [70]. MSCs have also shown to be able to differentiate into hepatocytes, further highlighting their applicability in liver-specific therapeutics [71]. However, so far, liver reconstruction using differentiated MSCs is in the early stages, and its potential has only been assessed in animal models [33]. Other tissues where MSCs have shown potential applicability include cornea, trachea, or skin regeneration, which include successful outcomes in clinical trials [33].

## 3. Cell-Implant Surface Interactions

Within our body, every tissue is characterized by a distinct ECM composed of a main backbone of fibrous proteins (i.e., collagens, fibronectin, elastin, and fibrillin) which provides tensile strength and elasticity to the tissue. Numerous glycoproteins and highly hydrated proteoglycans are also embedded within this fibrous ECM [72]. Proteoglycans accumulate and release growth factors and are also responsible for the stiffness of the connective tissue. Other tissue-specific constituents of the ECM include lipoproteins, phosphoproteins, and other post-translationally modified proteins [72].

Within the tissues, there are three different aspects of the ECM that are sensed by the cells, thus having an influence in modulating their behavior: (a) Biochemical aspects: the chemical nature of the constituting biomacromolecules, such as charge, hydrophobicity, and molecular composition; (b) mechanical aspects: the elasticity and resilience of the ECM; and (c) topographical aspects: the three dimensional (3D) architecture, including its micro- and nano-scale structure [72]. Therefore, the surface topography of the implanted scaffold will have an influence in regulating the cells behavior, including the SCs commitment towards defined cell lineages. At the microscale, the extent of cell spreading directly correlates to the total substratum coverage with ECM-proteins, regardless of the geometrical pattern [73]. In contrast, at the nanoscale, several reports are suggesting that the different cell types react specifically and in a differential manner to the topographical features in that range [72]. The different surface topographic elements at the nanoscale (i.e., roughness, patterns, and porosity) have the ability to promote changes in cytoskeletal organization, cell shape, focal contact formation, motility, differentiation, and gene expression [72]. Such a reaction to the substrate topography is partly attributed to adhesion-related mechanisms [74]. The cells can sense the different topographic features of the scaffold by means of integrin molecules, which are cell-surface transmembrane receptors having a central role in the transduction of “outside-in signaling” [75].

Once implanted into the host body, plasma proteins rapidly adsorb onto the surface of the scaffold before cell arrival. The chemical composition, topography or wettability of the scaffold, will have an impact on the type, amount and also conformation of the adsorbed proteins [74,76,77]. Next, the extracellular domains of the cell integrins will bind to the different peptide motifs of the adsorbed proteins (i.e., tripeptide Arg-Gly-Asp (RGD)). This integrin-ligand binding will result in the interaction and clustering of cytoplasmatic proteins, such as focal adhesion kinase (FAK), vinculin, or paxillin. These clusters of over 150 structural and signaling proteins, which are known as focal adhesion (FA) complexes, regulate intracellular signaling cascades (e.g., mitogen-activated protein kinases (MAPK) signals) [75,78]. Important processes, including proliferation, differentiation, or motility, are also associated with integrins [78,79,80,81].

Apart from integrin-ligand binding, it has also been suggested by several reports that, after the initial contact, the cells produce discrete nanolength projections (i.e., “nanopodia”) that transiently sense the surface to gain optimum anchorage and spreading [82,83]. Thus, the cells experience contact guidance from the different nanotopographical features gathering geometric information via nanoscale adhesion-localized structures [72,84]. Subsequent integrin-related FAs results in linkage to actin fibers (stress fibers) that are associated with cell spreading and enhanced cell movement [72,85]. On one hand, these acting stress fibers deform the nuclear membrane, which, in turn, results in the opening of nucleopores [78]. As a result of this nucleopore opening, there is enhanced mRNA transport to the cytosol, thus increasing protein translation. On the other hand, the cytoskeletal tension can promote the stretching of the cell membrane. This enhanced spreading of the cell membrane opens membrane-bound calcium ion channels allowing the transport of calcium ions to the cytosol [78]. This results in positive feedback with myosin light-chain kinase which translates into further cellular contractility [78]. Additionally, recent evidence is showing that cytoskeletal remodeling in response to surface topography can also affect nuclear morphology (nucleoskeleton arrangement), which will, in turn, result in chromosomal translocation and epigenetic DNA to regulate key genes that are involved in cell growth and functions [74,86].

## 4. The Effect of Substrate Topography

The fact that cell behaviors can be regulated by topographical cues from the underlying substrates was demonstrated as early as 1911 [87]. Now, it is widely acknowledged that, by contact guidance, cells can adjust their orientation and align along with the topographical features that they are cultured on. Since cells can respond to topographical cues down to 5 nm, the development of micro- and nanofabrication methods able to replicate the topographical cues of the cell niche in a controllable and reproducible fashion, has been crucial for the progress of the field [4]. Thus, we review and discuss the effect of the three main features of topography (i.e., roughness, surface patterns, and porosity) onto the cell-fate decisions of MSCs.

### 4.1. Substrate Roughness

Despite the enormous regenerative capacity of bone tissue, large bone defects that frequently accompany trauma, infection or tumor resection surgeries require therapeutic intervention. While autologous bone grafting is currently the gold standard therapeutic strategy, such a procedure has many drawbacks. For example, there is a limited supply of autologous bone, additional surgery is needed which leads to donor site morbidity, and the associated risk of infection [33,88]. Since alternative approaches are highly sought after, MSC-based bone regeneration has been explored [33,89].

#### 4.1.1. Ceramic-Based Scaffolds

Currently, the most common approaches to induce osteogenic differentiation of MSCs make use of growth factors (e.g., bone morphogenetic protein 2 (BMP-2)) or small molecular compounds (e.g., dexamethasone). However, the use of soluble factors entails some limitations and risks, such as an inherently short-term availability or the fact that they can have complex interactions with the medium. Thus, the use of biomaterials-based substrates with control over their different biophysical features (i.e., topography, matrix stiffness, and mechanical forces) has emerged as an alternative strategy to promote the formation of bone tissue in a more efficient and safe manner [90].

Roughness topography can be modulated to control the osteogenic differentiation of MSCs, thus being of particular interest for bone TE (BTE) [90,91,92]. Such an effect has been attributed to the fact that roughness topography may replicate the physical features left by osteoclast (OC) activity on the morphology of the bone surface during the process of bone resorption [92,93]. Thus, scaffolds with tailored surface roughness able to direct and continuously support the differentiation of MSCs, resulting on a robust and inexpensive approach have been extensively investigated [94]. For example, in a recent study, Yang and collaborators fabricated hydroxyapatite (HA)-based scaffolds with varying surface roughness [90]. HA is a very interesting material for BTE since it is the main inorganic component present in human bone. The authors prepared HA disks with varying average roughness (Ra) (ranging from 0.2 to 1.65 µm) and mean distance between peaks (RSm) (ranging from 89.7 to 18.6 µm) and evaluated the osteogenic differentiation of pre-seeded human BM-MSCs (hBM-MSCs). The expression levels of osteogenic genes (i.e., runt-related transcription factor 2 (RUNX2), osteopontin (OPN), alkaline phosphatase (ALP), and collagen type I (COLI)), together with the mineralization by conducting Alizarin Red staining (ARS), were assessed. ALP is a membrane-bound enzyme that hydrolyzes phosphate esters during mineralization, and is considered a marker of early osteogenic differentiation. In contrast, ARS is used to locate calcium deposits and it is an end point measure of matrix mineralization. Optimal osteogenic differentiation in the presence of osteogenic medium was obtained for scaffolds displaying a Ra from 0.7 to 1.0 µm and RSm ranging from 53.9 to 39.3 µm. Cell attachment, spread, and F-actin arrangement were also optimal for scaffolds displaying these particular Ra and RSm parameters, suggesting that surface topography might increase osteogenesis by affecting cell attachment and cytoskeletal tension [90]. Mechanistic studies were also conducted indicating that the expression of the nuclear transcriptional factor Yes-associated protein (YAP)/TAZ was involved in the osteogenic differentiation of the cells. This is not surprising since YAP/TAZ is known to be regulated by the actin cytoskeleton and to participate in controlling the osteogenic differentiation of MSCs [90,95,96].

Gradient surfaces are of particular interest in this context, since they allow for systematic studies by continuously varying a surface parameter per experiment. Faia-Torres and coworkers fabricated polycaprolactone (PCL)-based substrates with gradient Ra from the sub-micron to the micrometer range to the osteogenic differentiation of MSCs [92]. PCL was chosen as the material since it is a resorbable polymer that has already been approved for craniofacial bone implants by the United States Food and Drug Administration (FDA) [97]. The substrates, which were created by applying a hot-embossing replication technique, displayed Ra gradients from ~0.5 to 4.7 µm and RSm values gradually varying from ~214 µm to 33 µm. The results indicated that surface roughness displaying Ra and RSm values in the range of 2.1–3.1 µm and 71.1–48.1 µm, respectively, had the biggest impact on the osteogenic differentiation of human BM-MSCs. These Ra and RSm values resulted in a faster osteogenic commitment and stronger osteogenic expression as shown by the ALP values which were more than 10 times higher than the control material (tissue culture polystyrene substrate). Robust COLI deposition was also observed for such surface roughness. Interestingly, the optimal RSm values for these PCL-based scaffolds (i.e., in the range from 71.1–48.1 µm) are the closest ones to the reported RSm values found in native bones (i.e., ~40 µm) [92,93]. While this first work was conducted in the presence of growth factors, on a follow-up study, the impact of surface roughness on the osteogenic differentiation of MSCs was assessed in the absence of osteogenic inducers [94]. In this study, Ra and RSm values of ~1.53 µm and ~79 µm, respectively, supported the highest ALP and COLI expression, as well as the highest mineralization for human hBM-MSCs cultured in the presence of osteogenic inducers. In contrast, Ra and RSm values of ~0.93 µm and ~135 µm, respectively, supported the highest ALP and COLI expression and mineralization when using basal growth medium only. Importantly, a Ra of 0.93 µm was able to direct the differentiation of BM-MSCs towards the osteogenic lineage even in the total absence of cell-differentiating factors [94]. These results demonstrated, for the first time, that specific surface roughness on its own was able to induce a strong osteogenic differentiation of hBM-MSCs. It was hypothesized by the authors that such specific Ra values promoting the commitment of BM-MSCs towards the osteogenic lineage, might mimic the pits left by osteoclastic bone resorption. Surface roughness gradients have also been explored onto catecholic polyglycerol coatings, which were the highest formation of FAs and filopodia, as well as the highest cellular tension, was observed for a Ra of 278.64 nm [98]. Such a Ra value also enhanced the osteogenic differentiation of MSCs. Interestingly, substrates with a Ra of 1050 nm were able to maintain the phenotype of MSCs by minimizing cell adhesion.

#### 4.1.2. Titanium (Ti)-Based Scaffolds

Due to the load-bearing function of bone implants, metal materials have been widely explored in BTE. Ti is a particularly important material in this context. Ti is biocompatible, light-weighted but stronger than other metals, such as steel or cobalt alloys, which are also used as medical implants [99,100]. The excellent biocompatibility of Ti is attributed to the natural Ti dioxide deposited on its surface [99]. Although Ti-based implants have been widely employed as hip replacements, bone plates or in dental surgeries, pure Ti materials lack a good chemical bonding to the bone. As a result, about 5–20% of Ti-based implants fail in the early post-implantation period [101,102]. An efficient strategy to improve the biological performance of Ti implants is to implement topographical structures, including roughness. However, the reported studies show substantial discrepancies, a fact that can be attributed to the lack of a high-throughput strategy which could have been achieved by using surface roughness gradients [98]. For example, a first study conducted in 2009 showed how Ti surfaces with small Ra of 15 nm in height enhanced the adhesion and osteogenesis of MSCs as compared to Ras of 55 and 100 nm [103]. However, on a different report, Ras of 150 and 450 nm enhanced the osteogenic differentiation of MSCs as compared to Ra of 20 nm [104]. A different study showed no difference in terms of OBs adhesion was observed for Ti substrates with different roughness [105].

**Figure 1:**
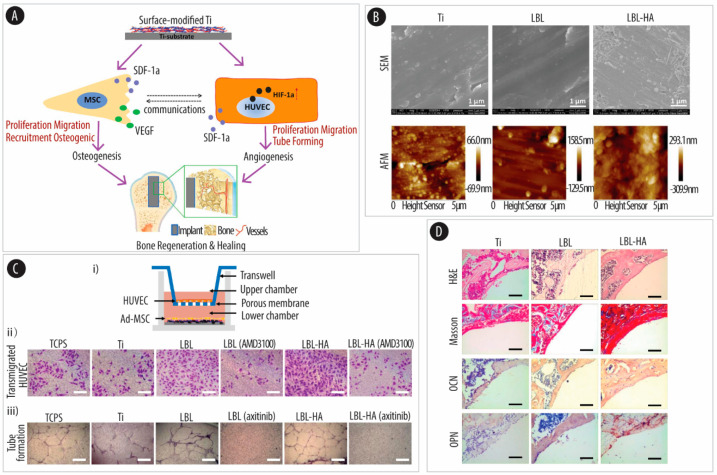
Multilayered coating of titanium (Ti) implants promoting coupled osteogenesis and angiogenesis in vitro and in vivo. (**A**) Schematic illustration of coupled osteogenesis and angiogenesis by surface-modified Ti substrates for enhanced bone healing. (**B**) Scanning electron microscopy (SEM) and atomic force microscopy (AFM) images of the Ti substrate, gelatin (Gel)/chitosancatechol (Chi-C) multilayer Ti-coated substrate (LBL) and the hydroxyapatite (HA) nanofiber-embedded LBL substrate (LBL-HA). (scale bar: 1 μm). (**C**) Effect of the substrates on the angiogenic factors secreted by adipose-derived mesenchymal stem cells (AD-MSCs) and enhanced regulation of human umbilical vein endothelial cells (HUVECs) functions. (i) Scheme of AD-MSCs and HUVECs co-culture system. (ii) Images of transmigrated HUVECs that were stained by crystal violet. LBL (AMD3100) and LBL-HA (AMD3100) groups were treated with C-X-C chemokine receptor type 4 (CXCR4) inhibitor AMD3100. (iii) Images of vasculogenic networks formed by HUVECs. For the LBL (axitinib) and LBL-HA (axitinib) groups, the vascular endothelial growth factor receptor (VEGFR) inhibitor axitinib was added to the co-culture system. (scale bar: 200 µm). (**D**) Rat histopathological detection of bone formation after implantation for 28 days. Hematoxylin and eosin (H&E) staining (scale bars: 200 µm), Masson staining (scale bar: 200 µm), osteocalcin (OCN) staining (scale bar: 100 µm), and osteopontin (OPN) staining (scale bar: 100 µm) of femoral tissue. Adapted from [101], with permission from Elsevier, 2018.

The impact of surface roughness employing Ti substrates has been extensively studied by the Cai group. The authors developed a sub-micrometer topography composed of nanosheet-pore structures onto pure Ti surfaces [101,106,107]. The surfaces, which were prepared by a simple vapor alkaline-treatment method, showed a Ra value of 0.05 µm with pore sizes ranging from 100 to 400 nm and depths ranging from 450 to 750 nm. In a first study, it was shown how these were able to promote the adhesion and osteogenic differentiation of MSCs as shown by protein adsorption, cell adhesion, differentiation and the expression of bone formation-related proteins (i.e., RUNX2 and Osterix (OSX)) [107]. Importantly, enhanced osteogenic differentiation of MSCs was also observed when cultured in medium without osteogenic inducers [107]. In a follow-up study, strontium ions (Sr^2+^) were incorporated into the substrates [106]. The aim was to combine both biochemical and biophysical cues to enhance the osteogenic differentiation of MSCs. Sr^2+^, which was incorporated into the Ti substrates via ion-change, is an inorganic bioactive element widely employed in BTE applications due to its ability in promoting proliferation, differentiation and mineralization of OBs [106,108]. The results revealed enhanced ALP expression and mineralization level, as well as up-regulation of RUNX2 and OSX genes for MSCs cultured onto the Sr^2+^-incorporating substrates. Additionally, the mRNA expression of ALP, COLI, OPN, and osteocalcin (OCN) was also dramatically enhanced for these substrates. Finally, the osteoinductive potential was also investigated in vivo using a rabbit femur defect model where a larger amount of new dense bone tissue was formed for the Sr^2+^-incorporating Ti substrates following 4 and 12 weeks post-implantation. Following on, the authors reported a new type of surface-modified Ti substrates by applying a multilayered coating of chitosan-catechol (Chi-C), gelatin (Gel) and HA nanofibers. The aim was to create a coating able to promote both osteogenesis and angiogenesis (Figure 1A) [101]. By scanning electron microscopy (SEM) and atomic force microscopy (AFM) measurements, the root mean square (RMS) surface roughness was evaluated, showing values of ~69.6 nm and ~23.5 nm for the multicoated and the uncoated Ti substrates, respectively (Figure 1B). Additionally, needle-like structures were observed for the multilayered substrates which was attributed to the incorporation of the HA nanofibers. The multilayered substrates were employed to evaluate the adhesion, morphology and recruitment of both AD-MSCs but also human umbilical vein endothelial cells (HUVECs). Due to the interrelation between osteogenesis and angiogenesis, studying the communication between OBs and endothelial cells is becoming a crucial aspect to understand the process of bone healing. Following co-culture with AD-MSCs, the multilayered substrates were able to significantly increase the invasion and vasculogenic network formation of HUVECs (Figure 1C). Significant enhancement in the stromal cell-derived factor 1a (SDF-1a) and vascular endothelial growth factor (VEGF) expression of AD-MSCs was also observed. These results were further corroborated in vivo following implantation for 7 days on a rat model. The histological examination of the rat femoral tissue showed a markedly increased angiogenic response together with the engraftment of autologous MSCs into the peri-implant tissue at an early stage of bone healing (Figure 1D). The only limitation of this study is that such a design, where both surface chemistry and roughness are coupled together, does not allow to discern which the individual contributions to the enhanced angiogenesis and osteogenesis.

Worth mentioning in the context of Ti implants is the fabrication of coating surfaces displaying osteogenic inducing activity but also antibacterial properties [109]. Inhibiting bacteria adhesion is an important feature since infection is a major problem often times leading to the failure of the implant [110]. Once a bacterial bio-film has been formed, it becomes very difficult to treat and it can lead to implant removal. Therefore, a promising strategy to reduce implant-associated infections would be to fabricate implants displaying topographical features able to minimize initial bacterial adhesion. Such a strategy was accomplished by Huang et al. [109]. By sandblasting and consecutive acid etching procedures, the authors were able to fabricate a hierarchical micro/submicron/nano-scale structure on a Ti substrate. The Ra of the substrates, which could be fine-tuned by the exposure time of the second acid etching step, ranged from 2.25 µm to 1.75 µm for the longest incubation time. By cell counting and SEM it was confirmed that the substrate with the lowest Ra (i.e., Ra of about 2.25 µm) displayed the lowest bacterial adhesion. Furthermore, such a substrate demonstrated enhanced proliferation and osteogenic differentiation of pre-OBs [109].

While the regulatory effect of substrate topography on the process of osteogenesis has been widely studied, the impact of implant surface topography on osteoclastogenesis or OCs function remains largely unexplored. In a pioneering study, the Boyan group explored the effect of microrough Ti surfaces on the communication among MSCs, OBs and OCs [111]. For that, the authors collected the conditioned media from MSCs and OBs that had been cultured on three Ti surfaces with different topographies: smooth, hydrophobic-microrough, and hydrophilic-microrough for 7 days. The collected conditioned media from was then used to culture OCs for 2 days, and the OCs activity was subsequently evaluated by quantifying the amount of released collagen followed by mRNA quantification. The results indicated that both MSCs and OBs were able to suppress OCs activity. The OCs activity was also influenced by the surface roughness of the Ti substrate. This study demonstrates, for the first time, that the implant surface properties have an effect in the communication among the different cells involved in the remodeling of primary bone.

All in all, these discussed examples demonstrate how, by fine-tuning the surface roughness of an implant, it is possible to influence the cell lineage decisions of MSCs. Thus, this strategy could potentially be employed in orthopedic applications as a robust and inexpensive alternative to the less safe soluble osteogenic inducers.

### 4.2. Substrate Patterns

Surface modifications with patterns at both micro- and nanorange have also demonstrated the ability to influence the commitment of MSCs towards a selected cell lineage [112]. This is of particular relevance for BTE applications since, by engineering implants with surface modifications (i.e., surface roughness or micro/nanopatterns), it will be possible to control the interaction between the scaffold and the host tissue [113].

#### 4.2.1. Ceramic-Based Scaffolds

Bioceramics, in particular, HA-based scaffolds, have been widely explored in this context. However, although 70% of the inorganic material of native bones is constituted by HA, HA-based scaffolds lack osteoinductive ability. This fact negatively impacts their repair capacity for large bone defects, non-unions and follow-up function restoration [114,115]. Thus, micro- and nanopatterned topographies have been implemented to enhance their osteoinductive properties. For example, HA-based scaffolds displaying nanopatterns fabricated with nanorods, have been evaluated. Specifically, the effects of orientation, spacing and diameter of the nanorods have been assessed and optimized [116,117,118,119]. The different studies have shown how quasi-upright HA nanorods are much more efficient in increasing the viability and proliferation of OBs as compared to the bent or parallel oriented counterparts [116]. The effect of the spacing between the quasi-upright HA nanorods was also evaluated for a range from 30 to 300 nm [117,118]. The results showed how a 70 nm interrod spacing promoted the highest osteogenic effect on pre-seeded MSCs, while also boosting the in vivo osteointegration of the scaffold. The impact of the nanorod diameter on osteogenesis was also evaluated keeping a constant interrod spacing of 70 nm [119]. From the three different studied nanorods (i.e., with 30, 70, and 150 nm in diameter), a 70 nm diameter was the best in terms of promoting the osteogenic differentiation of MSCs. Specifically, the 70 nm-diameter nanorods were able to direct the differentiation of MSCs towards the osteogenic lineage even in the absence of osteogenic supplements. Optimal osteointegration, evaluated by histological inspection employing Van Gieson’s staining, was also observed for the 70 nm-diameter nanorods. The biomechanical strength following integration of the bone-implant, which was assessed by a pull-out test, was also optimal for the 70 nm-diameter nanorods. The authors suggested that the 70 nm-diameter nanorods favored FA formation on the pre-seeded MSCs and the subsequent induced mechanotransduction [119].

Surface patterns employing nanorods constituted by bulk metallic glass (BMGs) have also been evaluated [120]. BMG is an interesting material since it can be easily molded both at the micro and nano length scale with good precision and high aspect ratios [120,121,122,123]. In contrast to conventional metals and alloys, BMGs can also be processed like plastics above their glass transition temperatures [124]. In a recent study, flat and nanopatterned BMG surfaces were assessed in the context of MSCs differentiation [120]. The nanopatterns, consisting in 200 nm-diameter nanorods fabricated by thermoplastic nanomolding, were able to direct the differentiation of MSCs towards the adipogenic lineage. MSCs seeded in the nanopatterned surfaces, displayed a rounded morphology while also forming few FA points. In contrast, MSCs seeded onto flat BMGs showed enhanced formation of FAs and a commitment towards the osteogenic lineage. The cellular localization of the YAP nuclear transducer was also evaluated. Assessing YAP localization in MSCs is important in this context since it is know that the presence of YAP in the nuclear region indicates osteogenesis while a loss of nuclear YAP takes place in MSCs undergoing differentiation towards the adipogenic lineage [120,125]. The results confirmed that, when cultured onto the nanopatterned substrates, mainly cytoplasmatic YAP could be observed while flat BMGs surfaces induced the nuclear localization of YAP. The authors also compared the osteogenic ability of flat BMGs and the grade 5 Ti substrate, showing a more pronounced differentiation for the former surface. Since BMGs have an elastic modulus in the GPa range, the authors speculate that BMG could be considered as an alternative to Ti in load-bearing surgical interventions.

**Figure 2:**
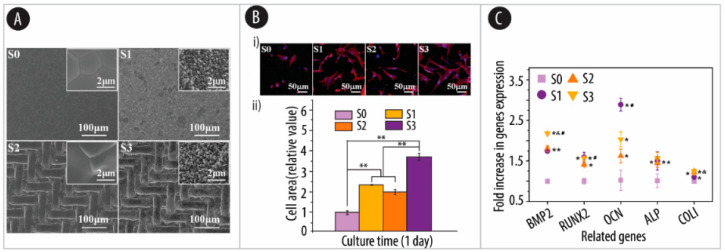
Micro-pattern and nano-topography of hydroxyapatite bioceramics stimulating the osteogenic differentiation of MSCs. (**A**) Field emission scanning electron microscope (FESEM) images of the control sample S0 with a smooth ceramic surface and the HA bioceramics with nanorod surface structure (S1), micropatterned surface structure (S2) and micropattern/nanorod hybrid surface structure (S3). The insets in the top-right corner of S0, S1, S2 and S3 correspond to high magnification images. (**B**) (i) Confocal images showing cytoskeleton of human bone marrow MSCs (hBM-MSCs) cultured on the samples S0–S3 for 24 h; (ii) Quantification of cell spreading area. * *p* < 0.05, ** *p* < 0.01. (**C**) Osteogenic genes expression of hBM-MSCs cultured on S0–S3 for 7 days. * indicates significant differences between S0 and the other samples, & indicates significant differences between S1 and the other samples, # indicates significant differences between S2 and the other samples, *p* < 0.05. Adapted from [112], with permission from Elsevier, 2018.

HA-based scaffolds with ordered micropatterns have also been explored. Using a micropatterned nylon sieve as a template, quadrate convex micropatterns of different sizes were fabricated [126]. With this method, the micropattern height and width could be easily regulated by tailoring the meshes of the nylon template. The differentiation of BM-MSCs was evaluated and the highest osteogenic response was observed for BM-MSCs cultured onto micropatterns with sizes close to that of a cell size. In a different study, the same group also evaluated the effect of three different surface topographies on the osteogenic differentiation of BM-MSCs [112,114]. Figure 2A shows SEM images of the three different patterns namely, nanosheets, nanorods and the hybrid of both nanorod and microrod (micro-nano-hybrid). The authors speculated that, since native bones display hierarchical features both in the micro- and nanorange, creating scaffolds combining both micro- and nano-structural features could result in additive or even synergistic effects [112]. The results, as shown by cell adhesion studies (Figure 2B), proliferation, and gene expression of osteogenic genes (Figure 2C, demonstrated a synergistic effect for the micro/nano hybrid structures on the osteogenesis of human BM-MSCs. Such a synergistic effect was attributed to the fact that the nanorod and micropattern structures resulted in the activation of different integrin subunits, BMP-2 downstream receptors and gap-junction related connexin 43 (Cx43) proteins. These facts indicate that micro- and nanostructures have different roles in regulating the fate of SCs. This is an important aspect that needs to be taken into account when designing biomaterials-based scaffolds for BTE. Importantly, in vivo studies were also conducted in a rat critical-sized calvarial defect model [114]. While the three different topographies resulted in enhanced new bone formation and mineralization as compared to scaffolds with flat surfaces, the micro-nano-hybrid combination was the structure promoting the best results both in vitro and in vivo.

Several other studies have also evaluated the ability of hierarchical micro/nano structured surfaces in stimulating the osteogenic commitment of MSCs [112,127,128,129]. For example, α-tricalcium phosphate (TCP) has been employed as a precursor to fabricate HA scaffolds with nanosheet, nanorod and a hierarchical micro/nano structure consisting of a hybrid of nanorods and microrods [129]. With such an approach, the authors were able to demonstrate that the hierarchical micro-/nano-topography surfaces not only significantly enhance cell attachment and viability of pre-seeded MSCs but also the ALP activity and the mRNA expression levels of both osteogenic and angiogenic markers. Follow up studies by the same group demonstrated that these hierarchical structures could significantly enhance the regeneration of new bone in a rat critical-sized calvarial defect model [128]. 

**Figure 3:**
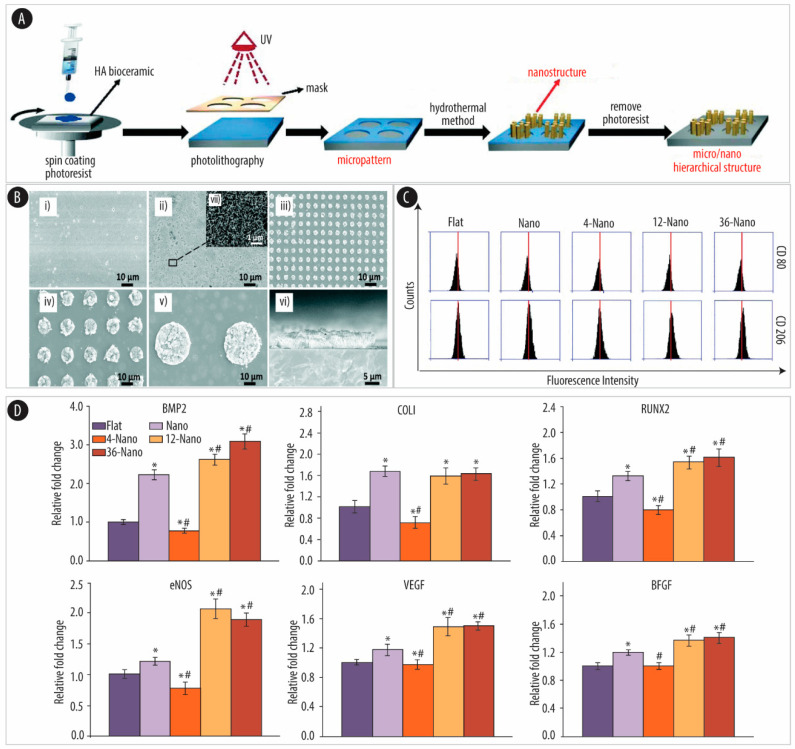
Micro/nano hierarchical hydroxyapatite (HA) stimulation of osteogenetic and angiogenetic differentiation via macrophage immunomodulation. (**A**) Schematic of the preparation of HA bioceramics with micro/nano hierarchical surfaces using photolithography and hydrothermal techniques. (**B**) SEM images of HA bioceramics with different sized micro/nano hierarchical structures. (i) Control sample with a flat surface (Flat group). (ii) Only nanoneedle surface (Nano-group). (iii) 4 µm micro-dot/nanoneedle hierarchical surface (4-Nano group). (iv) 12 μm micro-dot/nanoneedle hierarchical surface (12-Nano group). (v) 36 µm micro-dot/nanoneedle hierarchical surface (36-Nano group). (vi) Cross-section of the 36-Nano group. (vii) The corresponding high magnification image of the 36-Nano-group. (scale bar: 10 µm (i, ii, iii, iv, v), 5 µm (vi), 1 µm (vii)). (**C**) Fluorescence intensity of cluster of differentiation (CD)80 (top) and CD206 (bottom) of RAW 264.7 (mouse mononuclear macrophages) cells on HA bioceramics with different topographical surfaces. (**D**) RT-qPCR results of the osteogenic gene expression (bone morphogenetic protein 2 (BMP-2), collagen type I (COLI), and runt-related transcription factor 2 (RUNX2)) of hBM-MSCs and angiogenic gene expression (endothelial nitric oxide synthase (eNOS), vascular endothelial growth factor (VEGF), and vascular endothelial growth factor (BFGF)) of human umbilical vein endothelial cells (HUVECs) cultured in RAW 264.7 cell-conditioned medium. * indicates significant difference (*p* < 0.05) compared to the Flat group. # indicates a significant difference (*p* < 0.05) compared to the Nano group. Adapted from [127] with permission from the Royal Society of Chemistry, 2019.

In a very recent and original study, the effect of hierarchical micro/nano structured surfaces was evaluated, not only in the context of the osteogenic differentiation of MSCs but also in terms of macrophage response [127]. This is an important aspect since, the reaction of the host immune system, has a central role in mediating the performance of the implant [127,130]. The inflammation elicited by the implanted scaffolds, will inevitably affect the processes of wound healing and tissue remodeling. As such, the ability to promote a positive immune microenvironment would ensure the long-term success of the implant [131]. In this context, assessing the macrophages’ response is of particular interest since macrophages are one of the predominant immune cells in our body having a central role in the inflammatory response [127,132,133]. Depending on the microenvironment, macrophages polarize towards two main phenotypes, including pro-inflammatory (M1) and pro-healing (M2) polarization, which will inevitably affect the healing of the tissue [134,135,136]. The macrophages response was evaluated on HA-based substrates with three different and well-defined patterned hierarchical micro/nano structures (Figure 3A) [127]. By combining photolithography and hydrothermal techniques substrates with three circular micropatterns of 4 µm, 12 µm, and 36 µm in diameter willed with similar nanoneedle structures were fabricated (Figure 3B). Such a size range was chosen based on the sizes of the mouse macrophage cell line RAW 264.7 (of about 10 µm in diameter when fully spread). The proportion of M1- and M2-polarized macrophages depending on the studied hierarchical micro/nano structured surface was evaluated by measuring the fluorescence intensity signal of the M1-polarized (CD80) and M2-polarized (CD206) markers. The results, as shown by flow cytometry measurements, demonstrated that the structures of 12 and 36 µm-diameter induced much less M1 polarization and much more M2 polarization than the 4 µm-diameter or the flat structures (Figure 3C). While RAW 246.7 cells cultured onto 36 µm-diameter structures showed the highest fluorescence for CD206, the smallest 4 µm-diameter yielded the strongest fluorescent signal for CD80. These results were corroborated by qRT-PCR by measuring the expression of the pro-inflammatory gene CCR7, iNOS and TNF-α. Next, the effect of the immune response elicited by the macrophages on osteogenic and angiogenic differentiation of MSCs and HUVEC, respectively, was also evaluated. To do so, the expression of osteogenic and angiogenic genes in MSCs and HUVECs, respectively, was evaluated after being cultured in conditioned media collected from the structure-activated RAW 264.7 cells (Figure 3D). Interestingly, the results show significantly enhanced expression for all the osteogenic genes for the 12 and 36 µm-diameter surface structure-stimulated RAW 264.7 cells. In contrast, the 4 µm-diameter group showed significant inhibition as compared to the flat group. Similarly, the expression of the endothelial nitric oxide synthase (eNOS), VEGF and basic fibroblast growth factor ( BFGF) angiogenic genes was also up-regulated for HUVEC cells cultured in conditioned media from the 12 and 36 µm-diameter surface structure-stimulated RAW 264.7 cells. Thus, all in all, these results indicate that specific micro/nano hierarchical structures are also able to promote both osteogenesis and angiogenesis due to their ability in regulating the immune microenvironment. As such, this study provides a new perspective of how micro/nano hierarchical structures may promote bone regeneration by the manipulation of the macrophage’s polarization.

Bioceramics have also been used in combination with different biomacromolecules, such as collagen or chitosan, to prepare composite scaffolds to be used as bone implants [137,138,139,140]. Amongst them, silk fibroin (SF) appears as an interesting material for BTE due to its excellent mechanical and functional properties, low immunogenicity and good biocompatibility [137,141]. Thus, SF has been employed in combination with HA to render SF/HA composite scaffolds with excellent biocompatibility, osteoconductivity, and osteoinductivity [142,143]. MSCs were seeded onto such composite scaffolds which were fabricated by using air-plasma-treated SF films. Following in vivo implantation into a rat subcutaneous model, the air-plasma-treated SF/HA films promoted the most efficient formation of bone.

#### 4.2.2. Ti-Based Scaffolds

Surface patterns on Ti implants have also demonstrated the ability to significantly enhance the adhesion and osteoblastic differentiation of MSCs.

Research efforts have focused on constructing TiO_2_ nanostructures onto the implant’s surface to control SCs fate and improve implant performance. TiO_2_ is a native oxide formed on the Ti metal surface which bonds directly to the adjacent bone. In particular, many studies have been focused on TiO_2_ nanotube arrays fabricated by anodization onto Ti substrates [144,145]. Examples include the work by Lv et al., demonstrating that TiO_2_ nanotubes of 70 nm in diameter are the optimal size to promote the osteogenic differentiation of MSCs [146]. Oh et al., also showed how nanotubes ranging from 70 to 100 nm-diameter promoted a dramatic elongation of pre-seeded MSCs, which translated into cytoskeletal stress and osteogenic differentiation [14]. In contrast, smaller TiO_2_ nanotubes of about 30 nm-diameter did not promote the differentiation of MSCs [14]. Additionally, nanostructured surfaces consisting of a regular array of columnar TiO_2_ nanotubes have also shown the ability to promote osteoblastic cell differentiation [14,147,148], which then translated into enhanced bone apposition and fixation following implantation into rat tibias [147].

Research efforts have also focused on understanding the mechanistic aspects of the osteogenic differentiation of MSCs promoted by TiO_2_ nanopatterns. Hou et al. recently postulated that the such differentiation was mediated by the transient receptor potential vanilloid 4 (TRPV4) via the regulation of the nuclear factor of activated T-cells, cytoplasmic 1 (NFATc1) and wingless-related integration site (Wnt)/β-catenin signaling [149]. They conducted a study to elucidate whether calcium ions (Ca^2+^), which are the central element of calcified tissues, participate in the osteoblastic differentiation promoted by the nanotubes. To do so, MSCs were cultured onto Ti disks containing Ti nanotubes (TNT) with diameters of ~30 and ~100 nm (designated as TNT-30 and TNT-100, respectively) making use of polished Ti disks (PT) as the control. The authors chose to study TRPV4 since this ion channel is responsible for the intracellular transfer of Ca^2+^ [150]. To assess whether the TNTs promoted the osteogenic differentiation of MSCs via TRPV4, MSCs were cultured onto TNT-30, TNT-100, and PT for 7 days, and the expression of TRPV4, NFATc1, and Wnt/β-catenin, as well as their cellular distribution, was evaluated. The results showed that both TNT-30 and TNT-100 substrates promoted the expression and activation of TRPV4. Additionally, when inhibiting TRPV4 in MSCs cultured on TNT-100, reduced expression of osteoblastic genes and protein levels of NFATc1 and Wnt3a/β-catenin was also observed. As such, these results revealed that the osteoblastic differentiation of)BM-MSCs is mediated by TRPV4 [149].

Apart from nanotubes, TiO_2_ nanorods have also demonstrated the ability to influence the lineage commitment of MSCs [151]. In a first study, clustered TiO_2_ nanorods were fabricated on an acid-etched microstructured Ti surface employing hydrogen peroxide. By PCR, ALP expression and ARS, together with OPN and OCN immunofluorescence analyses, it was shown that such a topography was able to promote the osteogenic differentiation of pre-seeded MSCs. In a more recent study, a TiO_2_ nanorod array was fabricated employing a hydrothermal method using tetrabutyl titanate as a precursor [145]. The behavior of MSCs was evaluated and compared to a well-polished surface of TiO_2_ ceramic. Studies on the proliferation, adhesion, morphology, and differentiation of the pre-seeded MSCs demonstrated commitment towards the osteogenic lineage for the TiO_2_ nanorod array surface. In contrast, the control TiO_2_ ceramic with a smooth surface promoted the self-renewal of MSCs. Thus, overall, this study reveals an approach where, by using only different surface topographies, it is possible to control self-renewal or differentiation behaviors of MSCs.

#### 4.2.3. Polymeric Substrates

The use of nanopatterns onto polymeric substrates to direct the differentiation of MSCs was pioneered by Dalby and collaborators [152]. In such a pioneering study, the authors employed electron beam lithography (EBL) to fabricate ultra-precise nanotopographies onto a poly(methyl methacrylate) (PMMA) and demonstrated that the substrates displaying deliberately disordered pits in a square arrangement promoted the differentiation of MSCs into OBs [152]. In contrast, when employing nanopatterns displaying pits arranged in a square lattice symmetry, the results were different, and the pre-seeded MSCs were able to maintain their stem-cell phenotype and self-renewal for up to 8 weeks [153].

Differentiation of MSCs into adipogenic lineages by nanopatterns was also evaluated by Abagnale et al. [154]. In this study, microgrooved patterns were fabricated using polyimide (PI), which is a biocompatible, resistant, and long-lasting polymer [155,156,157]. The authors conducted a study employing a combination of 25 different structures with a systematic variation of the width of grooves and ridges ranging from 15 µm-sized structures down to the sub-micron range. By varying one specific parameter at the time, the authors aimed at understanding the impact of individual topographic features on cellular functions. The results showed that MSCs were aligned parallel to the main axis of the grooves for all the studied 25 patterns. In the presence of differentiation media and while keeping constant the groove-width (to 5 µm), 15 μm ridges were able to enhance adipogenic differentiation. In contrast, 2 μm ridges resulted in increased commitment towards the osteogenic lineage. This fact was attributed to the direct impact of the physical size of the ridges on cellular morphology. As such, while elongated morphology might enhance osteogenesis, a round morphology might drive the cells towards adipogenic lineage. Interestingly, when making use of nano-patterns, the results were different and PI substrates with a periodicity of 650 nm increased differentiation towards both osteogenic and adipogenic lineages. This fact was demonstrated by the up-regulation of both adipogenic (adiponectin (ADIPOQ), peroxisome proliferator-activated receptor gamma (PPARG), and fatty acid binding protein 4 (FABP4)) and osteogenic (secreted protein acidic and rich in cysteine (SPARC), secreted phosphoprotein 1 (SPP1), and bone gamma-carboxyglutamic acid-containing protein (BGLAP)) marker genes. Thus, these results suggest that the support of nanotopography on in vitro differentiation is not necessarily lineage-specific. Mechanistic studies were also conducted. Western blot fragmentation analysis did not show increased translocation of YAP to the cytoplasm for MSCs cultured on PI 650 nm as compared to flat PI substrates. This fact reveals that, in this case, YAP, which has been previously shown to modulate differentiation of MSCs [95], was not affected by nanotopography and is not relevant for this mechanotransduction.

Polymeric substrates in the form of nanofibers have been widely explored as scaffolds for TE applications. Nanofiber meshes fabricated by electrospun display the required flexibility in terms structure and composition which can be modulated to orchestrate specific cellular responses [158]. Polymeric nanofibers fabricated by electrospun have also been investigated in the context of BTE. Examples include the development of PCL-based nanofibrous scaffolds incorporating HA-NPs or β-TCP, which demonstrated the ability to direct the osteogenic differentiation of MSCs without the need for chemical osteogenic inducers [159]. In addition, incorporation of HA-NPs into poly(3-hydroxybutyrate-*co*-3-hydroxyvalerate) (PHVB) nanofibrous electrospun scaffolds have also been considered [160]. PHVB is a biodegradable, non-antigenic and biocompatible polyester and together with HA-NPs supported the commitment of MSCs towards the osteogenic lineage. Furthermore, the implantation of the PHVB/HA-NPs scaffolds into critical-sized rabbit radius defects, resulted in complete reparation as shown by the radiographic results [161]. PHBV/HA-NPs scaffolds with aligned and random-oriented nanofibers have also been prepared and compared. While no differences could be observed on bone growth following implantation into critical-sized rabbit bone defects depending on the fiber orientation, the mechanical properties of the repaired bones were different depending on the orientation of the fibers. Specifically, sixteen weeks after implantation, the rabbit’s radii were taken out and the mechanical properties were evaluated conducting stress-strain curves. The elasticity modulus of the repaired bone yielded values closer to the control group (i.e., normal radii of rabbits) for the scaffolds with aligned fibers. Thus, these results suggested that the PHBV/HA scaffolds with aligned nanofibers are more suitable for BTE applications as compared to the random-oriented counterparts [161]. The same group also conducted studies to assess the underlying mechanism of the spontaneous osteogenic differentiation of MSCs seeded onto PHVB/HA-NPs scaffolds [162]. By immunofluorescence staining after 3 days and 7 days of cell seeding, it was possible to demonstrate that Wnt/β-catenin signaling pathway, BMP- small mothers against decapentaplegic (Smad) signaling pathway and MAPK were involved in the osteogenic differentiation of MSCs. These results are not surprising since the Wnt/β-catenin signaling pathway, which has a central role in bone cell function, it is known to be involved in the cell responses to implant surfaces [163,164]. The BMP-Smad signaling pathway has also been reported to mediate the biological effects of the implant surfaces [165], while MAPKs are known to be key mediators of the cellular responses to a variety of extracellular stimuli [162,166]. Mechanistic investigations were also conducted by the Soleimani group [167]. The authors evaluated the effect of fiber orientation on the osteogenic differentiation of MSCs using a poly-L-lactide acid (PLLA) scaffold. The different nanotopographical cues (i.e., aligned versus random) had an effect on the osteogenic commitment of MSCs through the cross-regulation between microRNAs (miRNAs) and long noncoding RNAs. Additionally, by using a combination of computational and experimental approaches, the authors could identify a set of miRNAs that can efficiently regulate BMP signaling pathway through multi-target genes as accelerators of osteogenic differentiation.

Scaffolds constituted by nanofiber meshes fabricated by electrospinning are of particular interest for tendon and ligament (T/L) TE. T/L are constituted by a dense regular connective tissue composed primarily by COLI and collagen type III (COLIII) fibers [168]. These fibers consist of triple helix tropocollagen molecules which are hierarchically arranged in a multilevel creating parallel collagenous fibrils. This hierarchical architecture provides tendons and ligaments with exceptional axial tensile load-bearing capacity. However, following injury, tendons and ligaments have difficulties to heal. This is a consequence of a lack of vasculature in these tissues, which results in a suboptimal supply of essential reparative factors at the injury site [169]. When T/L heal by themselves, the initially formed healing matrix does not display the arranged hierarchical architecture but, instead, is constituted by randomly arranged collagen mainly composed by the smaller and less organized COLIII [170]. As a result of this marked decrease in the COLI and COLIII ratio, the tissue will experience a reparative phase, whereby the structural integrity between the torn ends will be restored. However, this reconstruction does not result in tissue regeneration, where the COLI to COLIII ratio is similar to that of the native tissues. Gradual remodeling of this initially formed healing matrix will take place over the course of a year, where the tissue will experience enhanced fibril alignment along the tissue length, as well as an increase in the COLI and COLIII ratio. However, despite the gradual alignment of the collagen fibrils over time, minor disorganization and abnormalities persist and the tissue never regains its original properties and functionality [169,171]. Approaches to repair T/L tears include suture, however this usually results in scar tissue and the subsequent shortening of the tendon. This tendon shortening permanently limits the motion range and, furthermore, it also leads to a high rate of injury reoccurrence [172]. A different strategy is to employ autografts or allografts to replace the injured tissue. However, such an approach involves the risk of donor site morbidity (for autografts) and disease transmission or rejection (for allografts). Alternatively, synthetic or natural ECM-derived grafts can also be employed as replacements. However, these grafts fail in replicating the mechanical properties of the native tissues [173]. Thus, there is a need for tissue-engineered T/L that better mimic the features of native tissue for improved repair. Topographical cell guidance has been investigated to create scaffolds with the ability to promote aligned cellular orientation for T/L applications [169].

First studies demonstrating the beneficial effects of culturing cells on aligned electrospun fibers for T/L TE include the work by Lee et al. [162]. The authors fabricated aligned polyurethane fibers and observed how human ligament fibroblasts cultured onto the fibers displayed a spindle-shaped morphology and oriented in the direction of the fibers. These aligned cells were able to secrete much more collagen as compared to randomly-oriented fibroblasts. The ligaments’ tensile strength has also been shown to improve when fibroblasts are aligned along the length [169,174]. Aligned fibers were also fabricated by the Ouyang group and demonstrated that, when aligned, PLLA nanofibers were able to regulate the orientation of MSCs and promote their differentiation into the teno-lineage [175]. Collagen threads have also been aligned electrochemically and evaluated in the context of tendon regeneration [176]. Pre-seeded MSCs oriented in a parallel manner to the aligned fibers and it was shown how this anisotropic orientation induced the tenogenic differentiation even in the absence of biochemical factors. Tong et al. replicated not only the physical topography but also the elasticity of the tendons’ ECM using nanoimprinting [177]. MSCs cultured on such scaffolds displayed shape and alignment similar to that of MSCs seeded onto the “longitudinally-cut” section of tendons. While, in this first study, no significantly enhanced tenomodulin (TNMD) expression could be detected, the results were different upon coating this nanoimprint topography with COLI and enhanced TNMD expression could be observed [178]. Thus, these results highlight the need for an additional signal to complete the full differentiation process towards a tenogenic lineage. Coatings with COLI-mimetic peptides have also been considered [158]. Using peptides mimicking a specific domain has several advantages over using natural adhesive proteins, such as COLI, fibronectin, or laminin. Limitations of natural proteins include a potential immunogenic response by the host, protein purification issues or the fact that proteins display multiple adhesion domains that may induce conflicting intracellular signals [179]. PCL meshes with an aligned topography were fabricated by electrospinning and coated with the glycine-phenylalanine-hydroxyproline-glycine-glutamate-arginine (GFOGER) motif of COLI [158]. Such a peptide was chosen since this domain (which corresponds to the residues 502–507 of the α_1_(I) chain of COLI) interacts with α_2_β_1_ integrin, thus mediating the adhesion and differentiation of OBs, as well as the mineralization of the matrix [158,180,181]. In this study, the in vitro migration and differentiation of MSCs as a result of both the functionalization with the peptide motif and orientation of the fibers was evaluated. The results showed how coating with the GFOGER peptide resulted in enhanced migration, proliferation, and osteogenic differentiation of MSCs. A further enhancement on cell migration was observed on the aligned fiber meshes as compared to the randomly-oriented ones, and the migration took place along the direction of the fiber orientation. However, no enhanced osteogenic differentiation could be detected as a result of the fiber alignment.

In addition, in the context of T/L TE, the Goh group fabricated a 3D scaffold consisting of aligned SF electrospun fibers (SFEFs) [169,182]. In a first study, it was demonstrated that, following seeding with MSCs, the aligned SFEFs promoted both cellular and ECM alignment [182]. Additionally, enhanced differentiation of MSCs towards the ligament fibroblast lineage was also demonstrated by the expression and production of ligament-related proteins (i.e., COLI, COLIII, tenascin-C, and TNMD). Due to the relevance of mechanical stimulation during the healing of tendons and ligaments [170], on a follow-up study, the effects of mechanical conditioning on the 3D aligned scaffolds were evaluated [169]. The authors hypothesized that mechanical loading was as important as the cellular alignment for the maintenance of an appropriate in vivo cellular behavior and matrix remodeling in the context of T/L regeneration [169]. As such, the biophysical effect of topography and mechanical stimuli on the tenogenesis of MSCs was investigated. To do so, mechanical stimulation of the scaffolds was achieved by rolling them up and loading them into the chamber vessels of a bioreactor where the ends of the scaffolds were used for anchorage. Next, both a 5% translational strain and a 90° rotational strain mimicking the multidimensional straining environment of the in vivo situation, were applied. Interestingly, the results, as shown by the enhanced production of T/L-related proteins, revealed that the introduction of mechanical forces accelerated and intensified the tenogenic differentiation of the pre-seeded MSCs. These findings are in agreement with previous studies showing an increase in the levels of intracellular Ca^2+^ in both tendon and ligament cells following mechanical stimulation [169,183,184]. Other reports have also shown how tendon cells respond to mechanical loads by releasing adenosine triphosphate [169,185]. Additionally, applying a cyclic strain intensifies the expression of ECM proteins while also inducing the assembly of ECM structures [169,186]. However, to the best of our knowledge, this is the first report on the combined and synergistic effect of aligned nanopatterns and mechanical stimuli in the context of T/L TE. Overall, these results suggest accelerated matrix deposition and remodeling process for the treatment group which will, in turn, improve the tensile properties.

Three-dimensional scaffolds constituted by braided polymeric microfibers have also shown promising mechanical properties for T/L TE applications [187]. Braided scaffolds are an interesting material since their mechanical properties can be fine-tuned by changing certain braiding parameters, such as the number of the braided fiber bundle, fiber diameter, or braiding angle. However, most of the reported braided scaffolds are constituted by fibers with a size in the micrometer range, which results in suboptimal biological activity. In contrast, 3D scaffolds consisting of braided fibers in the nano range will better mimic the dimensionality of native collagen fibrils [188], thus providing a more favorable environment for T/L tissue regeneration [187]. As such, Barber et al. pioneered the fabrication of hierarchical scaffolds for T/L TE based on braided nanosized fibers [187]. The scaffolds were fabricated by braiding 3, 4, or 5 aligned bundles of electrospun PLLA nanofibers and the tenogenic differentiation of pre-seeded MSCs was evaluated. The results showed how MSCs adhered and aligned in parallel to the length of the fibers experiencing also a realignment of the actin cytoskeleton. This is an important fact since, aligned actin stress fibers, have previously shown protection from mechanical loading while also mediating appropriate mechanotransduction signals within tendon fibroblasts [187,189]. When cultured in the growth medium, the different braided scaffolds supported the proliferation of MSCs enhancing their pluripotency as shown by the upregulation of key pluripotency genes (i.e., Oct3/4 and Sox2). In contrast, when the MSCs seeded scaffolds were cultured in differentiation medium containing fibroblast growth factor 2 (FGF-2), growth differentiation factor 5 (GDF-5), and BMP-2 as growth factors, downregulation of the three embryonic stem cell markers was detected. However, the differentiation medium influenced the commitment of the MSCs towards osteogenesis and away from the tenogenic lineage. This was shown by the upregulation of RUNX2 and the downregulation of Scleraxis (SCX). Interestingly, such a trend could be reversed following mechanical stimulation. Upon applying a cyclic tensile strain onto the MSCs-seeded scaffolds, significant upregulation of SCX gene expression was observed. Additionally, the MSCS-seeded scaffolds were able to produce tissue-specific ECM in the presence of both mechanical stimuli and differentiation medium. Altogether, these results highlight the importance of a suitable mechanobiological environment for scaffolds to be used in T/L TE applications.

**Figure 4:**
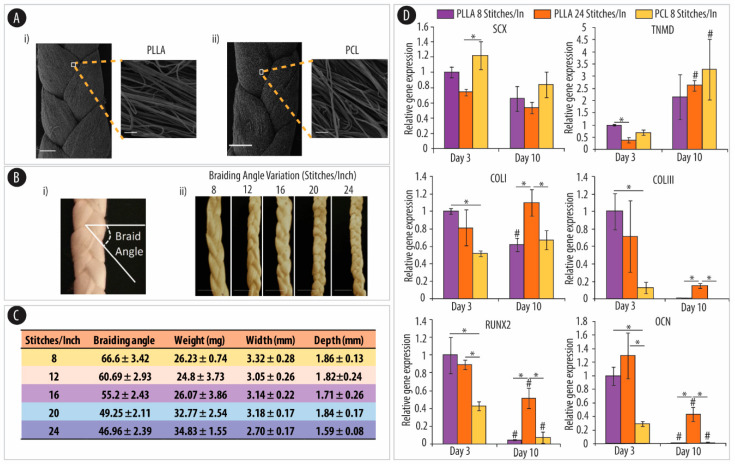
Properties of braided submicron fibrous scaffolds (BSMFSs) direct the tenogenic differentiation of human-induced pluripotent stem cell-derived mesenchymal stem cells (hiPSC-MSCs). (**A**) Representative SEM images of poly-L-lactide acid (PLLA) (i) and polycaprolactone (PCL) (ii) BSMFSs with insets showing a high magnification view of the fiber alignment. (scale bar: 500 µm, 10 µm (insets)). (**B**) Demonstration of braiding angle in a BSMFS (i) and macrographs of PLLA BSMFSs fabricated with different braiding angles by altering the number of bundle stitches per inch (8, 12, 16, 20, and 24) (ii) (scale bar: 5 µm). (**C**) BSMFSs with different braiding angles. (**D**) Quantitative RT-PCR results of mRNA expression of the tendon and ligament (T/L)-associated markers, Scleraxis (SCX), tenomodulin (TNMD), collagen type I (COLI), and collagen type III (COLIII), and the bone-associated markers, runt-related transcription factor 2 (RUNX2) and osteocalcin (OCN), at days 3 and 10. * denotes significant differences (*p* < 0.05; *n* = 3) between conditions at the same time point and # denotes significant differences (*p* < 0.05; *n* = 3) within the same condition between days 10 and 3. Adapted from [173] with permission from Elsevier, 2014.

In a follow-up study by the same group, the effect of the braiding angle onto the mechanical properties of the resulting braided electrospun nanofibers was evaluated [173]. The purpose of the study was to assess the effect of the mechanical properties of the resulting braided scaffold on the tenogenic commitment of MSCs. Interestingly, the authors employed iPSC-MSCs. iPSCs can be obtained by reprogramming many adult somatic tissues and, thus, they are an interesting cell source when MSCs are less available (e.g., from donors with increased age) [190]. In addition, iPSC-MSCs will render patient-specific cell lines for TE applications. PLLA and PCL fibers were prepared with different stitches per inch (SPI) (i.e., 8, 12, 16, 20, and 24) (Figure 4A,B) [173]. The different number of SPI resulted in braided nanofibers with different braid angles (ranging from 47 to 67°) (Figure 4C). The resulting fibers displayed different mechanical properties depending on the braiding angle, as shown by conducting stress-strain curves. Braided nanofibers with fewer SPI showed enhanced modulus and strength than those with more SPI. In addition, the mechanical evaluation demonstrated that PCL braided nanofibers were stronger and stiffer than their PLLA counterparts. The cell experiments were conducted by applying a cyclic strain stimulation, due to the relevance of mechanical loading in the in vivo cellular behavior of tissues and ligaments. It was demonstrated that PLLA was better than PCL in supporting cell adhesion, encouraging spindle-like morphology and inducing the characteristic tenogenic gene expression. These results are likely due to PCL’s hydrophobicity which will lead to less protein adhesion and the subsequent reduced cell attachment, proliferation, and differentiation [191]. Interestingly, while the nanofibers chemistry had an effect on the adhesion of iPSC-MSCs, the braiding angle played a major role in regulating their differentiation. Thus, braided nano fibers with fewer SPI promoted upregulation of tenogenic markers (i.e., SCX, TNMD, COLI to COLIII expression ratio) while downregulating the expression of the osteogenic ones (i.e., RUNX2, OCN) (Figure 4D). The authors speculated that, following application of a cyclic strain, the different mechanical properties of the scaffolds translated into varying mechanobiological forces onto the pre-seeded MSCs. This, in turn, by means of mechanotransduction, had an effect on the tenogenic differentiation of the cells. In a more recent study, aligned PCL- and PLLA-nanofibers with either a stacked or a braided architecture were fabricated and evaluated [192]. While both stacked [193] and braided [194] scaffolds have been fabricated both as tendon and ligament grafts, a direct comparison still remained to be conducted. The authors evaluated the effect of the macro-architecture on the mechanical properties and the subsequent tenogenic differentiation of MSCs. The results showed that while both types of scaffolds supported the tenogenic differentiation of MSCs, the braided constructs enhanced the upregulation tenogenesis to a greater degree than their stacked counterparts. As such, these results indicate that macro-architecture is also an important parameter that needs to be taken into account in T/L tissue engineering applications.

While the aforementioned reports have shown the ability to control the fate of MSCs in vitro by modifying the physical properties of the substrates, studies assessing whether the topography-induced effects observed in vitro can be also recapitulated in vivo, remain scarce. One of these few studies was reported by the Ouyang group [195]. The authors assessed whether the in vitro diverging differentiation pathways of MSCs promoted by aligned and randomly-oriented PLLA fibrous scaffolds could also be observed in vivo. SEM micrographs showed how MSCs were well attached to both types of scaffolds but displaying different morphologies. While MSCs cultured onto the randomly-oriented scaffolds were spread out showing a polygonal phenotype, when seeded onto the aligned scaffold, they displayed an elongated and spindle-shape morphology and were furthermore oriented parallel to the aligned fibers. The randomly-oriented fibers triggered the differentiation of the MSCs to the osteogenic lineage, as shown by the enhanced expression of osteogenic markers and the positive ALP staining. In contrast, increased tenogenic differentiation was observed for MSCs on the aligned substrate, as indicated by the enhanced expression of the tenogenic transcription factor gene SCX. Additionally, differences in the distribution of FA complexes, as well as the cytoskeletal organization of MSCs were observed depending on the alignments of the fibrous scaffolds. To get an in-depth understanding of the topographic induced differentiation of MSCs, the authors also conducted mechanistic studies. Specifically, the effects of cytochalasin D (cyto D) and the Rho kinase (ROCK) inhibitor Y-27632 on cytoskeletal reorganization and mechanotransduction were evaluated [195]. Addition of cyto D, a potent inhibitor of actin polymerization, onto the cell medium resulted in the cells to become rounded and no notable differences in morphology depending on the scaffold (i.e., aligned versus randomly-oriented) could be observed. Additionally, cyto D treatment downregulated the expression levels of both osteogenic RUNX2 and tenogenic SCX markers. Thus, cyto D could attenuate both the orientation-induced osteogenesis and alignment-induced tenogenesis. The addition to the cell media of Y-27632, which is an inhibitor of the myosin-generated cytoskeletal tension, eliminated cell elongation for MSCs on the aligned scaffold and reduced the projected surface area of MSCs in the randomly-oriented one. Thus, treatment with both cyto D and Y-27632, resulted in significant changes in cell morphology followed by loss of lineage commitment. These results suggest that the actin cytoskeleton is of high relevance for the lineage commitment of MSCs. Within the same study, both scaffolds were also evaluated in vivo on a rat Achilles tendon repair model. Following 4 weeks post-implantation, histological examination and immunohistochemical analysis showed that the aligned fibrous scaffolds promoted tendon-like tissue formation and yielded much more mature tendon as compared to the randomly-oriented ones. In contrast, for the randomly-oriented fibrous scaffold group, substantial chondrogenesis followed by tissue ossification and bone formation was observed at the implantation site. Thus, importantly, this study highlights the relationship between the topographic features of the scaffold and tissue formation and reveals possible risks of ectopic ossification induced by the design of the scaffolds in tendon-tissue repair applications.

Nanopatterned substrates have also been evaluated in the context of cartilage TE. Despite its simple appearance, treating adult cartilage tissue following injury remains a significant clinical challenge. This poor ability for self-regeneration is a consequence of several features: cartilage is an avascular tissue, has a low supply of progenitor cells and it also is a scarcely cellularized tissue with a single constituent cell type (chondrocytes) with a very poor proliferation ability [3,196]. However, articular cartilage is frequently damaged mainly due to trauma, inflammatory causes or degenerative joint diseases, such as osteoarthritis [197]. To date, the major treatment strategies for cartilage repair include microfracture (marrow stimulation) [198], autografts [199], and autologous chondrocyte implantation [196,200]. However, these methods have important limitations, which include lack of integration, graft instability, and calcification, as well as donor-site morbidity [196,197,201,202,203]. Therefore, TE approaches have emerged as a promising strategy to restore injured cartilage [197]. Due to the limited source of healthy donor cartilage, as well as the reduced proliferative ability of isolated autologous chondrocytes, the use of SCs has emerged as an alternative for generating the required cell numbers for viable cartilage repair [3,204]. MSCs offer an attractive cell source since they have the ability to differentiate into chondrocytes, have a high proliferation capacity and can also be easily extracted from donor BM [3,205,206].

**Figure 5:**
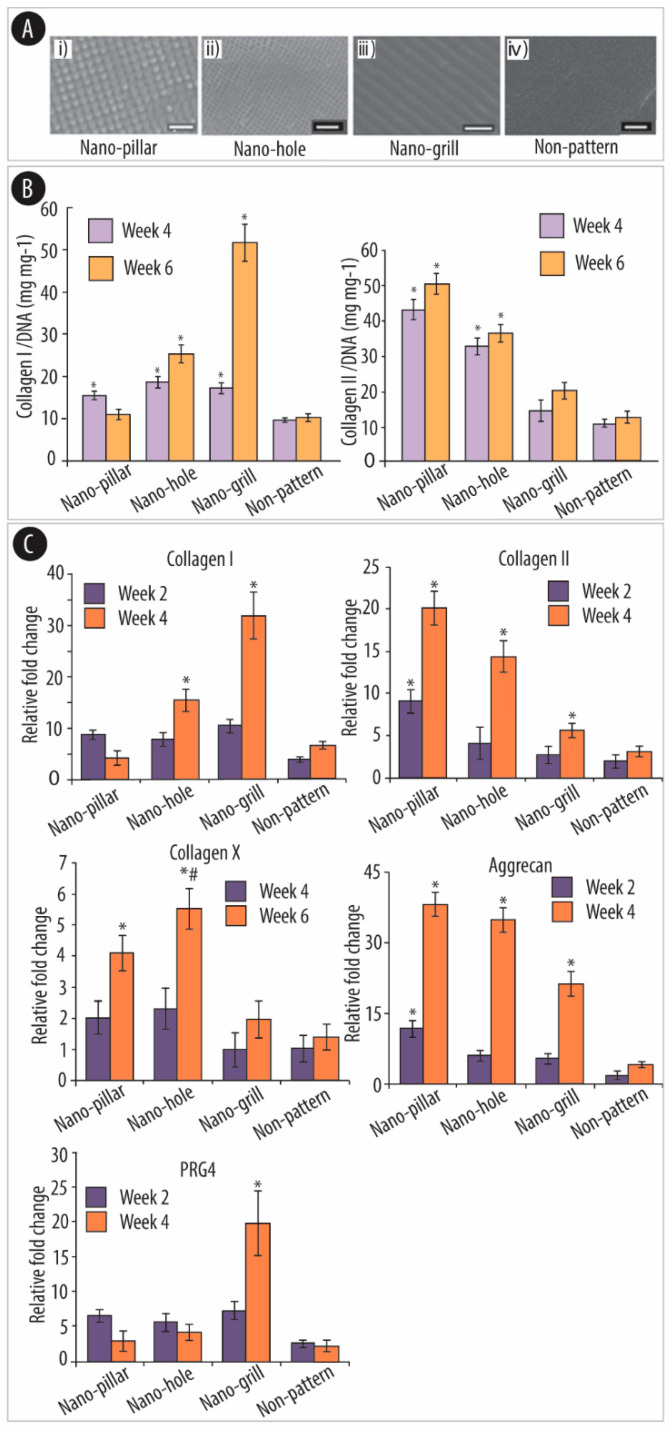
Substrate topography determines the fate of chondrogenesis from human mesenchymal stem cells (MSCs) resulting in specific cartilage phenotype formation. (**A**) SEM images showing the surface morphology of non-patterned and nano-patterned polycaprolactone (PCL) films with a chondroitin sulfate (CS) coating (scale bar: 1 µm). (**B**) Collagen I and II expressed by MSCs cultured on the different-patterned surface. The results were analyzed by enzyme linked immunosorbent assays (ELISA) and normalized to DNA at week 4 and week 6 of chondrogenic differentiation. * denotes *p* < 0.05, significant increase compared to non-patterned surface. (**C**) Real-time PCR analysis of mRNA expression levels of cartilaginous genes at weeks 2, 4, or 6 of chondrogenic differentiation normalized to their respective glyceraldehyde-3-phosphate dehydrogenase (GAPDH) expression and expressed as fold changes relative to undifferentiated MSCs. * denotes *p* < 0.05, significant increase compared to non-patterned surface. # denotes *p* < 0.05, significant different between nano-pillar and nano-hole surface at the same time point. Adapted from [3] with permission from Elsevier, 2014.

The Mauck group was one of the firsts in exploring the potential of MSCs for cartilage tissue engineering [207,208]. MSCs were used in the context of fibrocartilaginous tissues, which are tissues sharing the characteristics of both articular cartilage and fibrous tissues, such as tendon and ligament. Fibrocartilaginous tissues, such as the annulus fibrosus of the intervertebral disc and the knee meniscus, conduct mechanical roles that are essential for healthy joint function. In a first study, Baker and Mauk demonstrated the utility of MSCs for meniscus tissue engineering by showing how MSCs were able to align with the predominant nanofiber direction of a PCL scaffold and deposit fibrocartilaginous ECM [208]. Enhanced ECM deposition and improvement in tensile properties were observed for the aligned nanofibers as compared to the random-oriented ones [208]. The same group also reported a strategy for the engineering of annulus fibrosus, which is a multi-lamellar fibrocartilage of the intervertebral disk [207]. The study shows the fabrication of PCL-based scaffolds capturing the multi-scale structural architecture of the annulus fibrosus, which is a requirement to achieve the functionality of their native counterparts. Anisotropic nanofibrous laminates were created by electrospinning and, following MSCs seeding, they directed the deposition of a collagen-rich ECM mimicking the multi-lamellar hierarchical structure of the native annulus fibrosus. Additionally, such an architecture displayed similar mechanical properties than the native tissue after 10 weeks of in vitro culture. While this prior work was conducted making use of bovine MSCs, more recently, the same group employed human MSCs and evaluated their potential in fibrocartilage engineering [209]. Specifically, MSCs were harvested from the BM of patients undergoing total knee arthroplasty and, as a control, fibrochondrocytes were isolated from the meniscus of the same donors. Both cell types were cultured on aligned nanofibrous scaffolds and their ability to create a mechanically functional, fibrocartilaginous matrix was assessed over 9 weeks. Surprisingly, and in contrast to the results obtained using bovine MSCs, little matrix formation was observed for MSCs as compared to fibrochondrocytes taken from the same donor. These varying results could be attributed to the lack of a spatially-defined topography rendered by the electrospinning technique. Only by spatially controlling the patterned nanofeatures, it will be possible to conduct systematic studies on the chondrogenesis of MSCs. Thus, nanoimprinting has been also used to create specific nano-topographies and evaluate their ability to regulate the formation of cartilage tissue [3,210]. Nano-grill, nano-hole and nano-pillar patterns were chosen due to their resemblance to the topographical features of the cartilage collagen fibril (Figure 5A) [3,211]. According to the authors, approximately, a nano-grill topography resembles the parallel collagen fibers of the superficial and deep zone cartilage, nano-holes better mimic the pores among the randomly oriented collagen fibers while the nano-pillars represent the intersections of the random collagen fibers. PCL was also employed as the polymeric material to create a nanopatterned film. However, in this study, a chondroitin sulfate (CS) coating was deposited onto the substrate to provide chondro-inductive biochemical cues [3,212,213]. The results, as shown by quantifying the expression level COLI and collagen type II (COLII) by enzyme linked immunosorbent assays (ELISA) and the cartilaginous mRNA markers (COLI and COLII), demonstrated the ability of nano-pillar and nano-hole topography to enhance the differentiation of MSCs towards the chondrogenic lineage (Figure 5B). Additionally, the nano-pillar and nano-hole topographies facilitated the formation of hyaline cartilage as compared to the non-patterned substrate. In contrast, on a nano-grill topography, the MSCs experienced delayed chondrogenesis and were induced to fibro/superficial zone cartilage formation (Figure 5C) [3]. In a follow-up study by the same group, the combined effect of nano-topography and mechanical stiffness of the substrate was evaluated [210]. To do so, nano-grating and nano-pillar patterns were fabricated employing polyesters of varying stiffness [210]. PCL, polylactide (PLA) and polyglycolide (PGA) which have different mechanical stiffness (PCL<PLA<PGA) were thermally imprinted and coated with CS to obtain an even chondro-inductive surface across all polymeric substrates. The results demonstrated that substratum stiffness and topographical cues affected the phenotypic development at the earlier stage of chondrogenic differentiation. Additionally, different types of cartilage-like tissue were obtained depending on the topography and stiffness of the substrate. This is an important fact since, while previous studies of cartilage tissue engineering using MSCs resulted in homogeneous cartilage tissue structures [214], this has very little resemblance to the in vivo situation. In our body, articular cartilage can be classified into different zones (i.e., the superficial, middle, and deep zones), where both the chondrocytes morphology, as well as the ECM composition and structural arrangement, are greatly different. Additionally, the amount of proteoglycans, the alignment and complex interactions among collagen fibers and other matrix components, result in different mechanical properties that inevitably influence the overall function of articular cartilage [3,214,215]. Within this study, hyaline-like cartilage with middle/deep zone cartilage characteristics was obtained on softer surfaces with nano-pillar topographies. This was shown by the upregulation of aggrecan, CILP, COMP, and COLIX, which are middle/deep zone cartilage markers. In contrast, on stiffer and also nano-pillar substrates, MSCs generated constituents of hyaline/fibro/hypertrophic cartilage as shown by the high expression of COLII which was accompanied by low levels of COLI. Fibro/superficial zone-like cartilage could be obtained following culture on nano-grating surfaces of softer stiffness. This was demonstrated by the analysis of PRG4, which is a specific superficial zone cartilage marker. In contrast, stiffer nano-grating did not promote chondrogenesis.

### 4.3. Porosity

Scaffolds in tissue engineering are meant to act as a temporary ECM and, as such, they are expected to provide supports for cells and guide cell differentiation. Thus, since bioactive materials are designed to replicate the properties of in vivo environment, having an appropriate porosity and pore size are essential to control biological functions [74].

Thanks to recent developments in fabrication technologies, it is now possible to construct biomaterials with a well-defined pore structure that can be sensed by the cells of the host tissue (mainly SCs) and thus regulate cell fates during differentiation. The scaffolds pore structure, in terms of both porosity and pore size, provides space for the cell attachment and growth but also ensures the transport of nutrients, metabolites and waste products [74].

An appropriate porous structure of the scaffold is central to achieve an optimal osteogenic effect. This is not surprising given the porous structure of native bone. Specifically, there are two main types of bone: while the cancellous (or trabecular) bone shows a porosity ranging from 40–95%, the denser cortical (or compact) bone has a lower porosity of 5–25% [216]. Therefore, scaffolds for BTE require a porous architecture that will allow for the ingrowth of bone tissue and blood vessels while also ensuring bone oxygenation [74,217,218,219]. In particular, pore sizes of about 100 µm are considered beneficial for cell migration and nutrient transport [218]. However, prior reports have also found that pores larger than 200 µm were also able to promote vascularization and the formation of new bone [217]. Several studies have also found that smaller pores of around 50 to 100 µm were better for inducing endochondral ossification (i.e., osteochondral formation before osteogenesis) while, large pores (100–300 µm) promoted vascularization and were also beneficial for nutrient supply and waste removal [220,221,222]. Additionally, the porosity and connectivity among the pores are two important parameters. While high porosities of ~80% are regarded as optimal for the regeneration of new bone, cell ingrowth is also highly dependent on the degree of connectivity among the pores [220,221,222,223,224]. Additionally, recent studies are also indicating that interconnected pores are necessary to allow for the circulation of nutrients, waste and to promote the migration of cells to the center of the implant [225,226]. Thus, biomaterials scaffolds for BTE should exhibit both suitable pore sizes and porosity, as well as good interconnectivity among the pores [74,227,228,229].

The first studies assessing the effect of porosity on ceramic scaffolds date back to the early 1970s [230]. Hulbert et al. were able to demonstrate that pore sizes between 100 and 300 µm initiated bone formation, while a pore size range of 10 to 100 µm conditioned fibrous tissue or unmineralized osteoids [230,231]. Porous polymeric scaffolds were also developed in the 70s showing how bone ingrowth predominated when the scaffolds displayed a pore size around 450 µm [232]. Since these early studies, a wide range of pore sizes (from 20 to 1500 µm) have been investigated, however, the optimal pore size for OB activity is still controversial [233,234,235,236]. For example, one study demonstrated that OBs proliferation and bone formation on PLGA scaffolds was optimal for pore sizes ranging from 150 to 300 µm [237]. A different group also employing PLGA-based scaffolds showed how pore sizes in the range of 150 to 710 µm had no impact on the OBs attachment and proliferation [230,238]. Smaller pore sizes have also been investigated. For example, Akay et al. employed a highly porous polymeric foam (the so-called polyHIPE polymer) and, following the evaluation of different pore sizes, enhanced proliferation of OBs was demonstrated for small pores of 40 µm [239]. In contrast, scaffolds with larger pores (of about 100 µm) facilitated cell migration. However, no significant differences in the extent of mineralization or cell penetration depth could be observed depending on the pore size [239]. Collagen-based scaffolds have also been widely explored to identify an optimal pore size for BTE applications. In particular, the effect of the pore size on the preosteoblastic cell line MC3T3-E1 was evaluated employing collagen–glycosaminoglycan scaffolds [233]. In this case, optimal cell proliferation and infiltration were found for scaffolds with mean pore sizes larger than 300 µm. What is more, the ability of larger pores to facilitate cell infiltration was shown to counteract the favorable effect of the enhanced initial cell attachment surface areas provided by smaller pores. Thus, this study supports the hypothesis of previous reports highlighting the important role of pore sizes greater than 300 µm to promote osteogenesis [240]. The porosity and pore sizes in polymeric scaffolds have also been evaluated in the context of cartilage TE. Scaffolds consisting of biodegradable estane polymers displaying macropores in the range from 150 to 355 µm which are highly interconnected to micropores (<50 µm), have been shown to promote the ingrowth into fibrocartilaginous tissue preventing degeneration of the articular cartilage [230,241]. Studies evaluating the lineage commitment of MSCs depending on the scaffolds pore size have been conducted with polymeric substrates. Specifically, collagen-hyaluronic acid scaffolds with pore sizes of 94, 130, and 300 µm in diameter were evaluated [242]. The results showed that the largest (300 µm) pores promoted the best outcome in terms of chondrogenic differentiation. PLGA scaffolds with pore sizes in the range from 90 to 180 µm with a fibrin coating enhanced both the deposition of cartilage-specific ECM, as well as the chondrogenic differentiation, of pre-seeded MSCs [243]. iPSCs have also been studied in the context of regeneration and restoration of cartilage defects on 3D nanofibrous scaffolds with similar pore sizes [230,244].

Metals and alloys have been widely used as bone implants [245,246,247]. Thus, methods developed so far to fabricate porous metals include liquid state processing (e.g., direct foaming and spray foaming), solid-state processing, such as powder metallurgy and sintering of powders and fibers, electro-deposition, and vapor deposition [248]. While these methods offer control over both the shape and size of the pores by adjusting several parameters of the fabrication process, only a randomly organized porous structure can be obtained. In contrast, more sophisticated fabrication techniques, such as selective laser melting and electron beam melting, which are computer-controlled fabrication methods based on layer-wise manufacturing principles, render porous metals with a predefined external shape and internal architecture [248]. Similarly, as with ceramics or polymeric scaffolds, the literature evaluating the optimal pore size within metallic scaffolds still remains open to discussion. For example, Itala and co-workers, fabricated triangle-shaped Ti implants with pore sizes ranging from 50 to 125 µm [249]. Following implantation into rabbit femur, they found no clear lower limit of pore sizes for consistent bone ingrowth. The possible effect of microporosity, that is, small pores in the range of 0.5 to 10 μm on osseointegration, has also been evaluated [250]. Braem et al. fabricated microporous Ti implants that were placed in the cortical bone of rabbit tibiae. Following 2 and 4 weeks post-implantation, bone ingrowth could be observed throughout the porous Ti implant, highlighting that osseointegration of microporosities (<10 μm) could be achieved. Interestingly, when surface modifications inside the porous structure further reduced the interconnective pore size to a submicrometer level, the ingrowth of bone tissue was impaired. Extra-large pores in the range from 300 to 900 µm have also been investigated. Porous Ti scaffolds with pore sizes of 300, 600, and 900 µm were fabricated and implanted into rabbit tibias, and the results showed the highest bone ingrowth for the 600 and 900 µm-pore scaffolds [251].

**Figure 6:**
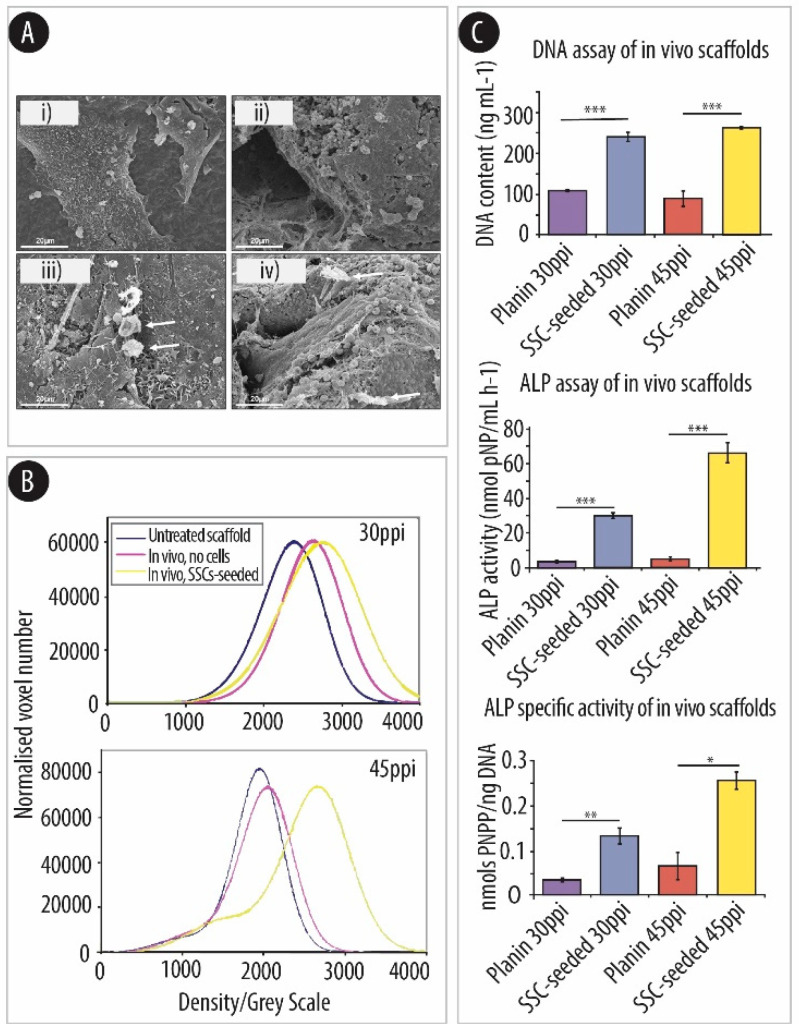
The effect of porosity of a biphasic ceramic scaffold on human skeletal stem cell (SSC) growth and differentiation in vivo. (**A**) SEM images of ex vivo scaffold with 30 pores per inch (30 ppi) (i), scaffold with 45 pores per inch (45 ppi) (ii), SSCs-seeded 30 ppi scaffold (iii), and SSCs-seeded 45 ppi scaffold (iv). Thick arrows indicate the SSC-like cells. (scale bar: 20 µm). (**B**) Density plots of scaffolds following 4 weeks in vivo growth with and without prior SSC-seeding. (**C**) Biochemical analysis of cellular material on the scaffolds following 4 weeks in vivo growth. * denotes *p* < 0.05, ** denotes *p* < 0.01, *** denotes *p* < 0.001 between the marked groups. Adapted from [254] with permission from Wiley, 2013.

Apart from the pore size, the level of porosity is an important parameter in bone regeneration since its envisioned that scaffolds with high porosity will facilitate the in vivo ingrowth of bone cells [230]. For example, Chan et al. evaluated the impact of different levels of porosity on the osteoinductive potential employing an ectopic bovine model [252]. Silicate substituted calcium phosphate scaffolds with porosities of 22.5, 32.0, and 46.0%, respectively, were implanted into the ovine parapsinalis muscle for 12 weeks. Histomorphometry analyses showed the largest amount of bone formation for the 46.0% porosity scaffolds with a 6.17 % increase in bone formation. In contrast, the lowest porosity level (22.2%) rendered only a 1.33 % increase in bone formation. Interestingly, bone formation was also observed in pores with a size smaller than 10 μm. Campion et al. also conducted studies with the same type of scaffolds in ovine critical-sized defect sites [253]. Their results also showed increased bone formation for the most porous scaffold but also more advanced neovascularization. Thus, these studies highlight the clinical importance of porosity for silicate-substituted calcium phosphate bone grafts. The effect of the level of porosity on the osteogenic differentiation of SCs has also been evaluated. Two groups of HA/TCP scaffolds with different porosity (i.e., 45 and 30 pores per inch, respectively) but controlled pore size were fabricated to evaluate the proliferation and osteogenic differentiation of skeletal SCs (Figure 6A) [254]. Following implantation of the seeded scaffolds into the rear flank of immunodeficient mice, the scaffold displaying 45 pores per inch, demonstrated the greatest increase in density (Figure 6B). While both scaffolds supported the proliferation of the skeletal SCs, the scaffold with the highest porosity was better in promoting the differentiation towards the osteogenic lineage (Figure 6C).

While a high level of porosity is envisioned to facilitate recruitment and penetration of cells from the surrounding bone tissue and its vascularization, thus benefiting bone ingrowth [248], it will also have a profound effect on the implants’ mechanical properties. This is of special interest for metallic implants. While metals and alloys have been widely used as bone implants, they are usually much stiffer than native bones. While cortical bones have elastic moduli ranging from 3 to 30 GPa, the elastic moduli of cancellous bones is only about 0.02–2 GPa [248]. In contrast, most current metallic implants used for bone regeneration have a much higher moduli. For example, a modulus of around 110 GPa and 210 GPa has been reported for Ti6Al4V and CoCrMo alloys, respectively [245,246,247]. Such a difference in stiffness between natural bone and metallic implants results in stress shielding which is a major source for bone resorption subsequently leading to the eventual failure of the implants [245,248]. An efficient method to circumvent stress shielding at the bone-implant interface, is to introduce controlled porosity in the materials [248,255]. Examples of porous metallic scaffolds to be used in BTE applications include cylindrical dental implants of cancellous structured Ti which were placed into a canine model. Following 8 weeks after the operation, more bone ingrowth could be detected for the implants with the highest level of porosity [256]. While a high level of porosity may be beneficial to reduce the elastic moduli of metallic implants, it might be detrimental for other less stiff materials. For BTE applications, a porous scaffold should also display appropriate mechanical properties (e.g., stiffness, strength, and toughness). Only scaffolds with satisfactory mechanical properties will be able to keep the shape following implantation in the body, while providing sufficient structural support during bone formation [225]. Therefore, the porosity of the scaffolds must be carefully tailored to achieve appropriate mechanical properties and biological performance [74]. As an example, Eqtesadi et al. evaluated the effect of porosity on the mechanical properties of 13–93 BG scaffolds. By immersing the scaffolds into simulated body fluid to mimic the in vivo degradation behavior, they demonstrated that the formed HA crystal on the porous scaffold surface was larger and more numerous than that on the dense scaffold surface. However, the presence of the porosity significantly diminished the scaffolds mechanical strength [257]. 

## 5. Conclusions and Outlook

The magic ability to regenerate injured or diseased human body parts to restore missing or lost function, has long been a dream of humanity. Biomaterials-based scaffolds in combination with MSCs represent a promising tool in regenerative medicine. Such an approach will instruct cell fate to regain lost tissue volume and function, thus improving patients’ quality of life.

Human MSCs have the ability of multi-lineage differentiation. Additionally, they possess the capability to self-renew over long periods of time, and they can be easily isolated with minimally invasive procedures. Although it has been over 50 years since their discovery, recent progress in extraction, culture and differentiation methods have paved the way for MSCs to become closer to clinical applications for tissue reconstruction.

During the course of tissue regeneration, the formation of new tissue is largely influenced by the physico-chemical cues of the microenvironment. Tissue cells reside in a complex milieu where they can communicate with each other while also responding to multiple stimuli provided by the surrounding matrix. As such, biomaterials-based scaffolds can provide multiple physico-chemical cues to modulate the growth and differentiation of MSCs and the subsequent formation of new tissue.

Traditionally, biochemical soluble compounds (i.e., growth factors) have been employed to regulate the differentiation of stem cells into a specific cell lineage. However, due to the limitations of these soluble factors (e.g., high costs, off-target effects, or challenges in obtaining optimal release kinetics), the focus has shifted towards adjusting the physical aspects of the stem cells environment. As such, biomaterials-based scaffolds with a well-defined topography have emerged as a powerful strategy. In this review, we have aimed to demonstrate how the different topographical features (i.e., surface roughness, patterns, and porosity) are an efficient method to control different aspects of cell behavior.

Despite the promising advancements presented herein, much work still remains to be conducted to successfully apply a biomaterials-mediated approach to guide MSCs for clinical use. While the studies conducted so far show the potential of the different topographical features to substantially modulate several aspects of MSCs, including proliferation, migration, alignment, differentiation, and even cell structure (e.g., cytoskeleton remodeling); such results have been observed on a case-by-case basis. Therefore, a universal correlation between the different topographical features and the subsequent cell response still needs to be established. To do so, future studies should focus on the interactions of cells and substrates comprehensively inspecting the biofunctions of the scaffold biophysical cues to unravel the underlying mechanisms. This is an important, but complicated, aspect due to the difficulty in reproducing the tissue structure in micro- and nanodimension. This translates into the need of developing new fabrication methods. In general, tissue formation takes place at multiple length scales. This hierarchical structure is responsible for many functional aspects of the tissue. Thus, it is envisioned that new or multiple combined fabrication techniques will be needed to engineer the scaffolds structure at molecular, nanoscale, microscale, and macroscale dimensions. Addressing these challenges will allow us to fabricate advanced implants with proper structural features able to provide the optimal cues to achieve the desired cell responses in vivo.
